# Organic acids induce plant defense responses and suppress root-knot nematodes in tomato via molecular mechanisms and molecular docking insights

**DOI:** 10.1038/s41598-026-56502-9

**Published:** 2026-06-10

**Authors:** Ibrahim A. Adss, Deiaa E. El-Habashy, Elsayed E. Hafez, Dina H. ELKobrosy, Ramy E. El-Ansary, Maher A. Hammad, Ghada M. El-Sayed

**Affiliations:** 1 Genetics department, Faculty of Agriculture, Damanhur University, Damanhur, Egypt; 2 Plant pathology department, Faculty of Agriculture, Damanhour University, Damanhour, Egypt; 3 Plant Protection and biomolecular diagnosis department, ALCRI, City of Scientific Research and Technological Applications, Alexandria, 21934 Egypt; 4 Zoology and Entomology Department, Faculty of Science, Al-Azhar University, Cairo, Egypt; 5 Department of Plant Protection, Faculty of Agriculture, Ain Shams University, Cairo, 11566 Egypt; 6 Microbial Genetics Department, Biotechnology Research Institute, National Research Centre, 33 El-Bohouth St, Dokki, 12622 Cairo Egypt

**Keywords:** Organic acids, Root-knot nematodes, Biocontrol, Peroxidase (POD), Polyphenol oxidase and (PPO) gene expression, Biochemistry, Biotechnology, Microbiology, Plant sciences

## Abstract

Root-knot nematodes (*Meloidogyne spp.*) are among the most destructive pathogens affecting tomato production worldwide, leading to severe yield losses and compromised plant health. This study evaluated the efficacy of six organic acids—benzoic, salicylic, citric, lactic, malic, and acetic acids—applied at three concentrations each, in managing *Meloidogyne incognita* infection in tomato cultivar “023.”Among all treatments, salicylic acid (SA) exhibited the strongest nematicidal activity, achieving 100% juvenile mortality within 72 hours and the highest inhibition of egg hatchability, even at the lowest concentration (0.01%). Benzoic and malic acid followed, showing strong nematocidal effects with moderate suppression of egg hatching, while citric and lactic acids displayed intermediate efficacy. Acetic acid was the least effective. Under greenhouse conditions, Salicylic acid alleviated nematode-induced growth loss through a significant reduction in root galling, egg masses, larvae, and egg counts, resulting in marked improvements in shoot and root biomass compared to the infected untreated control and other treatments. Citric and benzoic acids moderately improved plant growth, especially in root development, whereas lactic and acetic acids had minimal effects. At the molecular level, salicylic acid markedly upregulated the expression of peroxidase (POD) and polyphenol oxidase (PPO) genes, peaking at day 4 and remaining elevated through day 8. Citric, benzoic, and malic acids caused moderate induction, while lactic and acetic acids induced weak or transient expression. The expression pattern suggests dynamic and transient defense activation in response to organic acid treatments. Computational insights obtained through molecular docking simulations predicted that salicylic and benzoic acids may interfere with essential biological processes in *Meloidogyne incognita*. Both compounds exhibited the highest binding affinities toward several key pathogenicity-related proteins, including NAD(P)H oxidase, putative aspartyl protease, cytochrome c oxidase subunit 1, prefoldin-2, venom allergen-like protein Mi-vap-2, and protein disulfide-isomerase, complementing their in vivo nematicidal effects. Overall, salicylic acid at 0.01% emerged as the most potent and eco-friendly alternative for managing root-knot nematodes in tomato plants, combining strong nematicidal activity, enhanced plant defense responses, and molecular-level evidence of protein inhibition.

**Supplementary Information:**

The online version contains supplementary material available at 10.1038/s41598-026-56502-9.

## Introduction

Plant-parasitic nematodes (PPNs) are among the most destructive pests threatening global agriculture, causing annual crop losses exceeding $80 billion worldwide^1^. Among these, *Meloidogyne* species, commonly referred to as root-knot nematodes (RKNs), are particularly notorious due to their broad host range and high adaptability. They infect numerous economically important crops, including tomato (*Solanum lycopersicum*), a key horticultural crop cultivated in both open-field and protected systems. RKNs induce the formation of gall-like structures on roots, disrupting nutrient and water uptake, impairing plant growth, and predisposing plants to secondary infections by soilborne fungi and bacteria^2^. Once inside the roots, nematodes establish specialized feeding sites, resulting in severe morphological and physiological abnormalities such as gall formation, stunted growth, chlorosis, and yield reduction^3^. The severity of nematode-induced damage is often exacerbated by synergistic interactions with other rhizosphere pathogens. In tomato plants, RKNs interfere with the vascular system, increasing susceptibility to wilt pathogens such as *Fusarium oxysporum*^4^. Effective management of these nematodes remains a persistent challenge, primarily due to their subterranean lifestyle, broad host range, and rapid reproduction rate. Historically, chemical nematicides such as fenamiphos and oxamyl have been used to control RKNs. Although effective, these synthetic compounds pose considerable environmental hazards, including toxicity to non-target organisms, soil contamination, and increasing regulatory restrictions^5^. These limitations have prompted the search for sustainable and eco-friendly alternatives for nematode management. Among the most promising candidates are organic acids, which are naturally occurring, biodegradable compounds known to modulate plant defense mechanisms and/or directly inhibit nematode growth and reproduction.

Salicylic acid (SA), a phenolic phytohormone, plays a key role in activating systemic acquired resistance (SAR) and inducing the expression of pathogenesis-related (PR) genes. In tomato, SA treatment enhances defense-related enzymes such as peroxidases and Polyphenol oxidase, thereby reducing nematode penetration and development while improving root structure and plant resilience. Malic acid, a naturally occurring dicarboxylic acid involved in the Krebs cycle, also exhibits nematocidal properties by disrupting nematode energy metabolism and stimulating host defense responses. Yeon et al. (2022) reported that soil drenching with malic acid significantly reduced *Meloidogyne incognita* populations in tomato roots and increased phenolic compound accumulation and peroxidase activity. Similarly, citric acid, a tricarboxylic acid with antioxidant and chelating properties, has been shown to impair nematode viability by acidifying the rhizosphere and interfering with nutrient uptake^6^. It also enhances plant defense by stimulating systemic resistance responses. Benzoic acid, a simple aromatic carboxylic acid, exhibits broad-spectrum antimicrobial activity and has demonstrated efficacy in reducing nematode galling and reproduction through inhibition of egg hatching and juvenile motility^7^. Additionally, lactic acid, a fermentation-derived organic acid, has gained attention for its biocontrol potential, as it promotes plant defense enzyme activity and influences beneficial soil microbiota^8^.

Despite these findings, comparative studies evaluating the nematicidal potential of different organic acids and their physiological effects on tomato plants remain limited. Moreover, the specific mechanisms by which these acids modulate plant defense and suppress nematode infection are not yet fully understood. Therefore, the present study aims to evaluate the efficacy of selected organic acids—salicylic, malic, citric, benzoic, lactic and acetic acids—against *Meloidogyne incognita* infecting tomato plants. The research focuses on assessing their effects on nematode mortality, egg hatching, and plant biochemical defense responses, providing insight into their potential as sustainable alternatives to chemical nematicides.

## Materials and methods

### Nematode inoculum preparation

Nematode cultures were maintained by inoculating four-week-old tomato seedlings with *Meloidogyne incognita* in the greenhouse facilities of the Faculty of Agriculture, Damanhour University. After 45 days, egg masses were carefully removed from the galled roots of infected plants. Eggs were extracted using the sodium hypochlorite method described by Hussey and Barker^9^. The extracted eggs were transferred to Petri dishes containing fresh tap water and incubated at 28 °C for five days to allow hatching. Freshly hatched and active second-stage juveniles (J2s) were collected and concentrated by filtration through 500-mesh sieves. The suspension was adjusted to 2000 J2s per 5 mL using a counting slide (hemocytometer). Egg suspensions were also prepared and adjusted to 2000 eggs per 5 mL for subsequent experiments.

### Nematicidal assay of organic acids under laboratory conditions

#### Preparation of organic acid solutions

Six organic acids—salicylic, citric, malic, lactic, benzoic, and acetic acids—were employed in this experiment. All reagents were of analytical grade (≥ 99.99% purity) and obtained from Chemical Technology Co., Ltd. (China). Water-soluble acids were dissolved in distilled water with the addition of KOH to adjust the pH to approximately 6. Compounds with low water solubility were initially dissolved by dissolve one gram in 250 µL in of 95% ethanol then the volume was adjusted to 100 mL by adding distilled water to obtain the required stock concentrations that was put in a refrigerator for further treatments. For each organic acid, a stock solution (1 g/100 mL, w/v) was prepared, from which the working concentrations (0.01%, 0.05%, and 0.1% w/v; g/100 mL) were subsequently derived.

#### Juvenile mortality assay

In this experiment, an aliquot of double-distilled water containing approximately 200 freshly hatched second-stage juveniles (J2s) was prepared from the stock nematode suspension. This suspension was transferred into sterile glass tubes containing the appropriate volume of each organic acid, and distilled water was added to reach the desired working concentration. Each tube contained 10 mL of the mixture, including approximately 200 s-stage juveniles (J2s) and the specified concentration of the organic acid. To achieve a final concentration of 0.01%, 100 µL of the organic acid stock solution was added to the total volume of 10 mL; the same calculation was applied to prepare the other concentrations. Oxamyl was used as a positive control and applied according to the manufacturer’s recommended dosage.Tubes containing J2s in tap water were used as negative control. All tubes were incubated at 25 °C, and the numbers of live and dead juveniles were recorded after 24, 48, and 72 h using a binocular microscope. Nematodes showing active movement or a coiled (“winding”) body shape were considered alive, whereas immobile nematodes with straightened bodies were classified as dead. After 72 h of exposure, immobile nematodes were subjected to a revival test by transferring them into fresh tubes containing 1 mL of distilled water supplemented with a few drops of 1 M sodium hydroxide (NaOH). The mean mortality rate for each treatment was calculated and corrected using Abbott’s formula (Abbott, 1925) to account for control mortality:

$${\mathrm{mortality}}(\% ) = \frac{{{\mathrm{m}} - {\mathrm{n}}}}{{100 - {\mathrm{n}}}} \times 100$$

Where m is the mortality percentage in the treatment, and **n** is the mortality percentage in the control. All treatments were conducted in triplicate and repeated twice. The net mortality percentage was obtained by subtracting the percentage of nematode recovery in distilled water from the total mortality observed after 72 h.

#### Egg hatching inhibition assay

The hatching inhibition test followed the same procedure as the J2s mortality assay, except that an aliquot of distilled water containing approximately 200 *M. incognita* eggs selected from mature egg masses using sterile forceps from roots free of soil and were transferred to glass bottles with 2 mL was used instead of J2s. All tubes were maintained at room temperature (~ 25 °C) for 72 h to allow hatching. The number of hatched juveniles was then counted under a binocular microscope. After incubation, all treatments were transferred to fresh distilled water for 24 h to confirm the absence of recovery. The percentage of unhatched eggs was calculated as the hatching inhibition rate using Abbott’s formula :

$${\mathrm{Egg}}\;{\mathrm{inhibition}}(\% ) = \frac{{{\mathrm{m}} - {\mathrm{n}}}}{{100 - {\mathrm{n}}}} \times 100$$

Where **m** and **n** represent the percentages of unhatched eggs in the treatment and the control, respectively.

The net inhibition percentage was calculated by subtracting the percentage of recovery (hatched eggs in distilled water) from the inhibition percentage recorded after 72 h. All assays were performed in triplicate and repeated twice to ensure reproducibility.

#### Nematicidal assay of organic acids under Greenhouse conditions

In this experiment, the most effective concentrations of each organic acid (OA), as determined from the laboratory assays, were evaluated under greenhouse conditions. Uniform tomato seedlings (*Solanum lycopersicum* cv. “023”) were transplanted into plastic pots (2 kg capacity) filled with sterile sandy loam soil, which had been autoclaved at 121 °C for 1 h on two consecutive days. For OA-treated pots, each plant received a soil drench of the optimized concentration of the corresponding organic acid solution, as determined in the laboratory experiment. A volume of 500 mL of the lowest effective concentration was applied per plant, corresponding to 50 mg of each organic acid per plant.The treatments and their descriptions are presented in Table 1. Oxamyl pesticide was used as a reference treatment to compare the nematicidal performance of organic acids. Ten days after transplantation and OA application, each plant was inoculated with 5 mL of *Meloidogyne incognita* suspension containing approximately 2000 freshly hatched J2s. The inoculum was applied by removing 2–3 cm of topsoil around the plant base and distributing the nematode suspension evenly near the root zone. Each treatment included ten replicates. Organic acid applications were repeated every two weeks for a total of three treatments (spanning six weeks after nematode inoculation). Foliar sprays were applied to runoff as described by Yeon et al.^10^**.** Pots were arranged in a completely randomized design (CRD) under greenhouse conditions (27–32 °C, natural daylight) and maintained with regular irrigation. Fertilization was carried out weekly using a commercial fertilizer (Vascon 20–20–20; Farmers for Agricultural Development, Egypt) containing N (20%), P (20%), K (20%), and S (1.26%) at a rate of 2 g/L of water.

The experiment was concluded 42 days after nematode inoculation. Nematode reproduction was evaluated by recording the number of galls, egg masses per root system, eggs per 5 g of root tissue, and *Meloidogyne incognita* juveniles per 250 g of soil. Roots were carefully removed from the soil and thoroughly washed under running tap water to eliminate soil residues. To enhance the visibility of galls and egg masses, roots were immersed in 0.015% Phloxine B solution for 20 min following the method of Daykin and Hussey^11^, and then rinsed to remove excess stain. Egg numbers per egg mass were estimated by randomly collecting 15 egg masses from 5 g of roots and shaking them in 1% NaOCl for 3 min to release the eggs. The resulting suspension was filtered through 200- and 500-mesh sieves (75 and 26 μm) to remove debris and recover eggs^9^. Eggs were suspended in 100 mL of water, and counts were performed from 1 mL subsamples using a light microscope at 10× magnification. The density of second-stage juveniles (J2) in soil was determined using a series of sieves followed by a modified Baermann extraction, and juveniles were counted under a stereomicroscope^12^.

Additionally, plant growth parameters, including shoot and root length, fresh weight, and dry weight, were measured to assess the overall impact of organic acid treatments on plant performance. In parallel, non-infected plants without ant treatment to be used as negative control for measuring the relative genes (PPO and POD) expression.

**Table 1:** Greenhouse treatments for controlling *Meloidogyne incognita* infection in tomato plants.

Treatment code	Description
T1(positive control)	Infected plants without acid treatment
T2 (negative control)	non-infected plants without any treatment
T3 (reference treatment)	Pesticide (Oxamyl)
T4	Salicylic acid
T5	Citric acid
T6	Malic acid
T7	Lactic acid
T8	Benzoic acid
T9	Acetic acid

### Statistical analysis

Data were analyzed using two-way ANOVA (acid treatment × nematode infection) with SAS 9.4 software, considering both treatment and time as factors. Significant differences between means were determined using Tukey’s HSD test at a *p *value ≤ 0.05 (Tukey. 1949).

### Influence of treatments on peroxidase (POD) and Polyphenol Oxidase (PPO) gene expression in tomato roots

#### qRT-PCR experiments

Quantitative PCR was performed using a Rotor-Gene 6000 instrument (Qiagen, USA) in a total reaction volume of 25 µL. Each 25 µL qRT-PCR reaction contained 12.5 µL of 2× QuantiTect SYBR^®^ Green RT Mix (Fermentas, USA), 1 µL of each primer (10 pmol/µL), 1 µL of cDNA template (50 ng), and 9.5 µL of RNase-free water. Amplification was performed in a Rotor-Gene 6000 real-time PCR system (Qiagen, USA) under the following thermal cycling conditions: initial denaturation at 95 °C for 10 min, followed by 40 cycles of denaturation at 95 °C for 15 s, annealing at 60 °C for 30 s, and extension at 72 °C for 30 s, with a final extension at 72 °C for 10 min. Relative gene expression levels were calculated according to the comparative Cq (ΔΔCq) method^13^. The ΔCq value was determined as the difference between the Cq of the target gene and that of the reference gene (ΔCq = Cq_target − Cq_reference). The ΔΔCq value was then obtained by subtracting the ΔCq of the control sample from that of each treatment (ΔΔCq = ΔCq_sample − ΔCq_control). Relative expression levels were expressed as 2^(−ΔΔCq). Expression data were normalized against the 18 S rRNA reference gene, and the expression level of the untreated control plants at each time point was set to one (baseline). All qRT-PCR experiments were performed with three biological replicates for each treatment. Data are presented as mean ± standard error (SE), where SE = SD/n (*n* = 3).

**Table 2:** Sequences of primers used in quantitative real-time PCR (qRT-PCR) analysis.

Gene	Primer sequence		References
POD	Forward	5′-GCTTTGTCAGGGGTTGTGAT-3′	Jogaiah et al.^14^
	Reverse	5′-TGCATCTCTAGCAACCAAC-3′	
PPO	Forward	5′- CATGCTCTTGATGAGGCGTA-3′	Goel et al.^15^
	Reverse	5′-CCATCTATGGA CGGGAAGA-3′	
18 S rRNA	Forward	5′-GTGCATGGCCGTTCTTAGTTG-3′	Jayanna and Umesha^16^
	Reverse	5′-CAGGCTGAGGTCTCGTTCGT-3′	

### In silico analysis

#### Selection of target proteins of *M. incognita* and molecular modelling

In this study, several key target proteins essential for the growth, development, and pathogenicity of *Meloidogyne incognita* were selected for molecular modeling and docking analysis. The selected proteins included Cytochrome C oxidase subunit 1 (UniProt: A0A193H5K2), Putative aspartyl protease (UniProt: F0UYY7), Prefoldin-2 (UniProt: A0A0U1ZVZ2), NAD(P)H oxidase (UniProt: Q4JJA9), Venom allergen-like protein Mi-vap-2 (UniProt: A7 × 975), and Protein disulfide-isomerase (GenBank: XP_035445168.1). The corresponding FASTA sequences of these proteins were subjected to BLAST analysis to assess query coverage and percent identity (PI). Based on the results, proteins with > 30% PI were categorized for homology modeling, while those with < 30% PI or lacking suitable template structures were assigned for ab initio modeling. The proteins Cytochrome C oxidase subunit 1, Prefoldin-2, and Putative aspartyl protease possessed experimentally validated and computationally predicted 3D structures available from AlphaFold. Their corresponding PDB files were downloaded from the UniProt database for further analysis. Protein Disulfide Isomerase 1 was modeled using a homologous template structure (PDB ID: 4EKZ_A), selected based on its high query coverage and percent identity values. For target proteins with low sequence similarity or lacking homologous templates—specifically NAD(P)H oxidase and Venom allergen-like protein (Mi-VAP-2)—structural models were generated using I-TASSER. The top-ranked models were selected according to their C-scores, indicating the best structural confidence^17^. The finalized and energy-minimized protein models were then employed for molecular docking to evaluate their interactions with potential nematicidal organic acid ligands. For validation the protein models, validation was performed with computational servers, including VERIFY3D^18^ and PROCHECK^19^.

#### Molecular docking against target proteins

For molecular docking analysis, the two-dimensional (2D) structures of the selected organic acid ligands were retrieved from the PubChem database in SDF format. Hydrogen atoms were added to the 3D structures of the target proteins using AutoDock Vina’s MGL Tools^20^. Ligand structures were first converted to mol2 format using Open Babel^21^, and subsequently to pdbqt format with AutoDock Tools for compatibility with docking simulations. The active-site residues of each target protein were predicted using the COACH server^22^. Docking simulations were then performed using AutoDock Vina v1.0, which generated ten possible binding conformations for each ligand. The optimal docking pose was selected based on the lowest binding energy (highest negative value), indicating the most stable interaction. The resulting protein–ligand interactions were visualized and analyzed using BIOVIA Discovery Studio Visualizer (version 2021, Dassault Systèmes, San Diego, CA, USA). For each ligand, ten docking poses were examined, and the conformation exhibiting the strongest binding affinity was chosen for detailed study. The 2D interaction diagrams were generated using the *“Show 2D Diagram”* tool, while the *“Show Distance”* option was employed to visualize hydrogen bond distances and other interaction metrics. The H-bond surface receptor representation was also used to clearly illustrate the ligand-binding regions within the protein structures.

#### Samples collection and RNA extraction

In the greenhouse experiment, root samples were collected from three plants (three biological replicates) per treatment at 0, 2, 4, 8, and 12 days after inoculation with *Meloidogyne incognita* (J2s). The expression levels of the POD and PPO genes were analyzed using quantitative real-time polymerase chain reaction (qRT-PCR). Total RNA was extracted from 50 mg of fresh tomato root tips ground in liquid nitrogen using the RNA Isolation Kit I (BioTeke Corporation, China), based on the guanidinium isothiocyanate method, following the manufacturer’s protocol (Maxim Biotech Inc., USA). RNA purity and concentration were determined using a NanoDrop One spectrophotometer (Thermo Fisher Scientific, USA). Complementary DNA (cDNA) was synthesized from 1 µg of total RNA using an oligo(dT) primer, dNTPs, and M-MLV reverse transcriptase (Fermentas, USA) according to the standard procedure. The tomato 18 S rRNA gene was used as an internal reference (housekeeping gene) for normalization^23,16^. Gene-specific primers used in the analysis are listed in Table 2.

## Results

### Nematicidal assay of organic acids under laboratory conditions

This experiment was conducted twice, each with three replicates. Table 3 presents the effects of various organic acids (OAs) against the second-stage juveniles (J2s) and egg hatchability of *Meloidogyne incognita*. Noticeable variations were observed both among the different organic acids and across their concentrations at 24 h, 48 h and 72 h after treatment. Salicylic acid showed the highest nematicidal efficacy in both early (24 h and 48 h) and later (72 h) stages after treatment. At a concentration of 0.01%, it achieved 64.63% mortality after 24 h, 73.21% after 48 h, and reached 100% mortality after 72 h. Regarding egg hatchability, 0.01% salicylic acid inhibited egg hatching by 62% after seven days. Benzoic acid followed salicylic acid in effectiveness. Its concentrations of 0.05% and 1% showed nearly similar nematicidal patterns, causing 61.08% and 71.11% mortality after 24 h and 48 h, respectively, and 100% mortality after 72 h. Both concentrations also produced about 59.8% inhibition of egg hatching after seven days. The remaining four organic acids also demonstrated nematicidal activity against J2s and egg hatching, though with varying degrees of effectiveness. When comparing the lowest tested concentration (0.01%) for each acid, the order of efficacy was: citric > malic > acetic > lactic acid. Moreover, J2s that hatched from OA-treated eggs died within five days of incubation. Oxamyl served as the reference treatment to evaluate the relative efficacy of the tested organic acids. It exhibited a pronounced inhibitory effect on egg hatching, achieving 82% suppression after seven days. However, its nematicidal activity against J2 juveniles was comparable to that of most organic acids assessed in this study. To better clarify the results, the effects of identical concentrations of different organic acids (OAs) were compared. At the lowest tested concentration (0.01%), salicylic acid (SA) was the most effective treatment, showing greater efficacy than the corresponding concentrations of other OAs in increasing *Meloidogyne incognita* juvenile (J2) mortality and inhibiting egg hatching under laboratory conditions. The effectiveness of the tested OAs was evaluated based on the minimum concentration required to achieve significant nematode suppression, with consideration of cost-effectiveness and the potential to reduce phytotoxicity. Notably, SA exhibited a concentration-dependent paradox, where 0.01% SA resulted in higher J2 mortality than 0.05% and 0.1% after 24 h of exposure; however, this variation diminished over time, as all three concentrations caused 100% mortality with prolonged incubation of 72 h. A similar trend was observed for the other OAs, except lactic acid, whose efficacy increased markedly with increasing concentration.

**Table 3:** Effect of different organic acids and their concentrations on *Meloidogyne incognita* juvenile (J2) mortality and hatching inhibition under laboratory conditions.

Treatments	Concentrations	Mortality%24 h	Mortality %48 h	Mortality %72 h	Hatching%7 day	Reduction% hatching 7 days
Oxamyl		58.75 ± 1.83^ab^	78.36 ± 0.82^a^	100.00 ± 0.00^a^	14.25 ± 0.63^i^	82.08 ± 1.57^a^
Salicylic acid	0.01%	64.63 ± 0.80^a^	73.21 ± 0.83^ab^	100.00 ± 0.00^a^	30.19 ± 0.02^h^	62.04 ± 0.80^b^
	0.05%	58.02 ± 0.82^ab^	70.71 ± 0.72^abcd^	100.00 ± 0.00^a^	33.35 ± 0.75^fgh^	58.07 ± 0.78^c^d
	0.1%	58.02 ± 0.63^abc^	73.21 ± 0.62^ab^	100.00 ± 0.00^a^	32.36 ± 0.60^gh^	59.31 ± 0.59^bc^
Citric acid	0.01%	47.66 ± 0.84^cde^	64.07 ± 0.82^bcd^	95.83 ± 1.61^ab^	41.73 ± 1.39^cde^	47.54 ± 0.88^f^
	0.05%	40.36 ± 0.83^e^	68.10 ± 0.80^abcd^	100.00 ± 0.00^a^	41.37 ± 0.79^cdef^	47.98 ± 0.64^f^
	0.1%	46.35 ± 0.52d^e^	71.96 ± 1.73^abcd^	100.00 ± 0.00^a^	37.47 ± 0.49^defgh^	52.89 ± 1.8^e^
Malic acid	0.01%	51.21 ± 0.58^bcd^	60.86 ± 1.23^bcd^	86.41 ± 0.66^bc^	45.32 ± 0.65^cd^	43.02 ± 2.07^g^
	0.05%	59.62 ± 0.65^ab^	72.40 ± 1.42^abc^	95.13 ± 2.05^ab^	33.98 ± 0.58^efgh^	57.28 ± 0.68^cd^
	0.1%	52.26 ± 0.63^bcd^	63.75 ± 1.46^bcd^	92.03 ± 0.68^ab^	41.54c ± 0.72^def^	47.78 ± 0.68^f^
Lactic acid	0.01%	46.49 ± 1.00d^e^	42.73 ± 1.43^e^	51.08 ± 1.46^d^	68.34 ± 2.07^b^	14.08 ± 1.50^h^
	0.05%	51.52 ± 1.99^bcd^	60.18 ± 1.97^cd^	90.07 ± 1.48^ab^	44.02 ± 0.63^cd^	44.66 ± 2.29^g^
	0.1%	59.91 ± 0.56^ab^	67.91 ± 2.24^abcd^	97.56 ± 1.40^a^	34.68 ± 0.57^efgh^	56.40 ± 0.70^d^
Benzoic acid	0.01%	45.76 ± 0.04^de^	68.93 ± 1.67^abcd^	96.88 ± 0.79^a^	40.15 ± 0.84^cdefg^	49.53 ± 0.79f
	0.05%	60.94 ± 1.24^ab^	71.21 ± 0.83^ab^	100.00 ± 0.00^a^	31.97 ± 0.04^gh^	59.81 ± 0.83bc
	0.1%	61.08 ± 1.08^ab^	71.11 ± 0.73^abcd^	100.00 ± 0.00^a^	31.99 ± 0.89^gh^	59.79 ± 1.75bc
Acetic acid	0.01%	58.5 ± 0.48^6ab^	58.70 ± 1.41^d^	79.74 ± 1.26^c^	45.90 ± 0.43^c^	42.29 ± 0.51^g^
	0.05%	53.85 ± 1.67^abcd^	66.65 ± 1.65^abcd^	90.93 ± 0.05^ab^	40.20c ± 0.14^defg^	49.46 ± 1.61^f^
	0.1%	59.55 ± 1.43^ab^	66.11 ± 1.44^abcd^	98.37 ± 0.74^ab^	37.59 ± 1.25^defgh^	52.74 ± 2.23^e^

Treatments means sharing the same letter are not significantly different, while different letters denote significant differences according to the statistical test (*p* ≤ 0.05). Statistical comparisons were made among treatments within a single column. Each mean value came from three replicates.

### Nematicidal assay of organic acids under greenhouse conditions

The results regarding the minimum concentration of OA were applied and guided to be applied in greenhouse experiments. Regarding the effects of applications of organic acids, the recorded data indicates that the percentage reduction of the nematode reproductive parameters increased in case of salicylic acid. The data presented in Table 4 show that all tested organic acids significantly reduced *Meloidogyne incognita* infection parameters compared with the untreated control. The positive control recorded the highest number of galls (420), egg masses (349), juveniles in soil (501), and total eggs (134,308), confirming severe nematode infection. Among the organic acids, salicylic acid exhibited the strongest nematicidal effect, reducing the number of galls, egg masses, juveniles, and total eggs to 60, 48, 105, and 17,654, respectively. Citric, malic, acetic, and benzoic acids also caused noticeable reductions in nematode parameters, though to a lesser extent, while lactic acid showed the lowest efficacy among the organic acids tested. The synthetic nematicide oxamyl produced the most pronounced effect, almost completely suppressing nematode infection with only 2 galls, 1.5 egg masses, 3.25 juveniles, and 675 total eggs. Statistical analysis confirmed significant differences among treatments (*p* < 0.05), highlighting the potential of salicylic acid as a promising natural alternative to chemical nematicides.

**Table 4:** Effect of different organic acids on *Meloidogyne incognita* infection parameters under greenhouse conditions.

Treatment	Number of galls/roots	Egg-masses/roots	J2s/250 g Soil	Total eggs/5 g roots
Positive control	420 ± 12.11^a^	349 ± 8.31^a^	501 ± 14.76^a^	134,308 ± 53.56^a^
Oxamyl	2.00 ± 0.20^f^	1.50 ± 0.19^e^	3.25 ± 0.40^e^	675 ± 11.78^d^
Salicylic acid	60 ± 5.92^e^	48 ± 1.20^d^	105 ± 9.4^d^	17,654 ± 29.98^c^
Citric acid	134 ± 5.50^d^	113 ± 9.36^c^	201 ± 12.79^c^	31,471 ± 22.69^b^
Malic acid	161 ± 6.34^c^	131 ± 8.22^c^	228 ± 22.67^bc^	32,521 ± 25.19^b^
Lactic acid	192 ± 3.57^b^	169 ± 8.98^b^	247 ± 12.78^b^	39,613 ± 23.74^b^
Benzoic acid	157 ± 10.74^cd^	125 ± 6.80^c^	218 ± 13.90b^c^	31,501 ± 51.87^b^
Acetic acid	167 ± 6.98^bc^	137 ± 10.75^bc^	213 ± 14.98^bc^	36,245 ± 221.70^b^

Treatments means sharing the same letter are not significantly different, while different letters denote significant differences according to the statistical test (*p* ≤ 0.05). Statistical comparisons were made among treatments within a single column. Each mean value came from three replicates.

### Effect of different organic acids on shoot and root growth parameters of tomato plants infected with *Meloidogyne incognita* under greenhouse conditions

The impact of different organic acids on the growth parameters of tomato plants infected with *Meloidogyne incognita* is presented in Table 5. Significant variations were observed among treatments for both shoot and root traits. The positive control (infected untreated plants) recorded the lowest shoot fresh weight (19.75 g), shoot dry weight (4.50 g), and root parameters, indicating the negative effect of nematode infection on plant growth. In contrast, the negative control (uninfected plants) exhibited the highest growth performance, with shoot fresh and dry weights of 61.75 g and 13.75 g, respectively, and a root fresh weight of 17.25 g.

Among the treated groups, plants exposed to salicylic acid and oxamyl showed the greatest improvement in growth, with shoot fresh weights of 54.25 g and 54.75 g, respectively, and corresponding shoot dry weights of 12.00 g. These treatments also enhanced root development, yielding root fresh weights of 15.75 g and 13.00 g and root dry weights of 5.00 g and 3.80 g, respectively. Citric acid also positively influenced plant growth, producing relatively high shoot and root weights compared with the other organic acids.

Benzoic, malic, and acetic acids resulted in moderate increases in both shoot and root growth, whereas lactic acid exhibited the lowest enhancement among the tested organic acids. Although differences in shoot and root lengths were not statistically significant (*p* > 0.05), all organic acid treatments improved plant vigor compared with the untreated positive control. Overall, salicylic acid proved to be the most effective organic treatment in mitigating nematode-induced growth suppression, closely approaching the efficacy of oxamyl.

**Table 5:** Effect of different organic acids on shoot and root growth parameters of tomato plants infected with *Meloidogyne incognita* under greenhouse conditions.

Treatment	Shoot system			Root system		
	Shoot fresh weight (g)	Shoot dry weight (g)	Shoot length (cm)	Root fresh weight (g)	Root dry weight (g)	Root length (cm)
Positive control	19.75 ± 0.40^c^	4.50 ± 0.71^c^	17.50 ± 0.97^f^	7.50 ± 0.96^f^	2.28 ± 0.40^d^	8.75 ± 0.99^g^
Negative control	61.75 ± 0.24^a^	13.75 ± 1.05^a^	24.50 ± 1.02^ab^	17.25 ± 1.04^a^	5.48 ± 0.90^a^	18.75 ± 1.11^a^
Oxamyl	54.75 ± 0.54^ab^	12.00 ± 0.99^ab^	23.75 ± 0.40^b^	13.00 ± 1.35^c^	3.88 ± 0.13^c^	12.50 ± 1.06^e^
Salicylic acid	54.25 ± 0.57^ab^	12.00 ± 0.67^ab^	25.00 ± 1.24^a^	15.75 ± 0.99^b^	5.00 ± 0.48^ab^	15.75 ± 1.2^bc^
Citric acid	37.50 ± 0.49^abc^	8.00 ± 0.92^abc^	22.00 ± 0.69^c^	13.50 ± 0.69^c^	4.05 ± 0.43^bc^	15.00 ± 0.99^c^
Malic acid	35.00 ± 0.43^abc^	7.50 ± 0.41^abc^	20.25 ± 1.03^d^	11.50 ± 0.99^e^	3.50 ± 0.16^c^	11.25 ± 1.80^f^
Lactic acid	23.00 ± 0.87^c^	4.75 ± 0.33^bc^	20.25 ± 1.12^d^	12.00 ± 1.22^de^	3.63 ± 0.27^c^	16.00 ± 2.80^b^
Benzoic acid	35.75 ± 0.24^abc^	7.75 ± 0.703^abc^	20.75 ± 1.11^d^	11.75 ± 0.84^e^	3.40 ± 0.58^c^	13.50 ± 2.1^d^
Acetic acid	24.25 ± 0.59^bc^	5.25 ± 0.45^bc^	18.75 ± 0.24^e^	12.75 ± 0.95^cd^	3.85 ± 0.40^c^	15.50 ± 1.69^bc^

Treatments means sharing the same letter are not significantly different, while different letters denote significant differences according to the statistical test (*p* ≤ 0.05). Statistical comparisons were made among treatments within a single column. Each mean value came from three replicates.

### Effect of organic acids on the expression of the POD and PPO defenses gene in tomato plants infected with *Meloidogyne* incognita

#### Expression of POD gene in response of nematode infection and OAs application

**Fig. 1:**
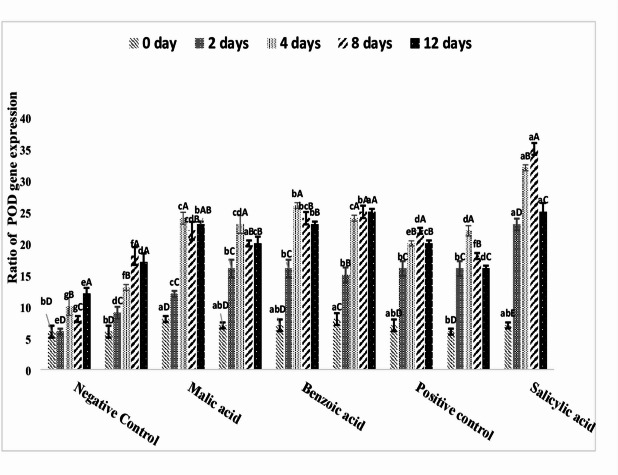
Effect of organic acid treatments on *POD* gene expression in tomato roots at different time intervals post-inoculation with *Meloidogyne incognita*. Data are presented as mean ± SD (*n* = 3). Error bars indicate the standard deviation of the mean. Treatments means sharing the same letter are not significantly different, while different letters denote significant differences according to the statistical test (*p* ≤ 0.05). Statistical comparisons were made from interaction between treatments and time. Each mean value came from three replicates.

The effect of different organic acids on *POD* (peroxidase) gene expression in tomato plants infected with *Meloidogyne incognita* is presented in Fig. 1. The results showed that *POD* gene expression increased progressively after treatment, reaching its peak between 4 and 8 days, followed by a slight decline by day 12. Among the treatments, salicylic acid induced the highest *POD* gene expression, with a peak ratio at day 4 and increased progressively at day 8, indicating a strong activation of the plant’s defense response. Benzoic, citric, and lactic acids also caused notable upregulation of *POD* expression, though at lower levels than salicylic acid.

Oxamyl treatment exhibited a moderate increase in *POD* expression, similar to the pattern observed with malic and acetic acids, while the positive control (infected untreated plants) showed only a slight induction throughout the experiment. The negative control (uninfected plants) maintained the lowest expression levels, confirming that the elevated *POD* activity in treated plants was a response to nematode infection and organic acid application.

Overall, salicylic acid proved to be the most effective inducer of *POD* gene expression, followed by benzoic and citric acids, suggesting their strong role in enhancing the antioxidant defense mechanism of tomato plants against *M. incognita* infection.

### Expression of PPO gene in response of nematode infection and OA application

**Fig. 2:**
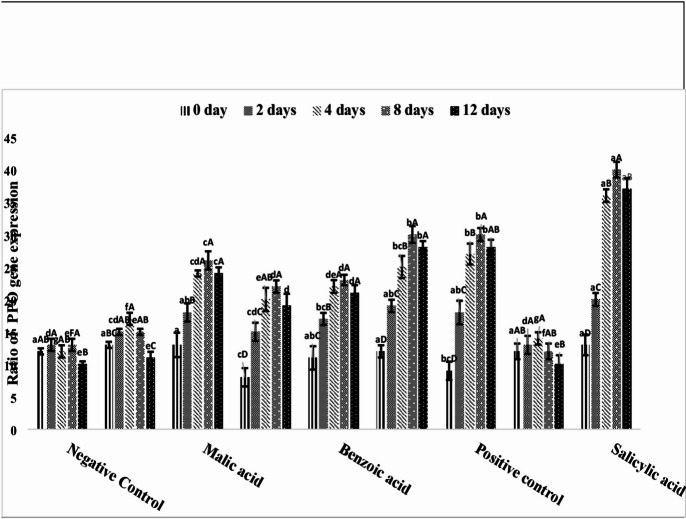
Effect of organic acid treatments on *PPO* gene expression in tomato roots at different time intervals post-inoculation with *Meloidogyne incognita*. Data are presented as mean ± SD (*n* = 3). Error bars indicate the standard deviation of the mean. Treatments means sharing the same letter are not significantly different, while different letters denote significant differences according to the statistical test (*p* ≤ 0.05). Statistical comparisons were made from interaction between treatments and time. Each mean value came from three replicates.

Figure 2 illustrates the effect of different organic acids on the expression level of the *PPO* (polyphenol oxidase) gene over time (0, 2, 4, 8, and 12 days). The results compare plant responses to each acid treatment with both negative and positive controls. Among all treatments, salicylic acid induced the strongest upregulation of *PPO* expression. Its expression peaked sharply on day 4, followed by a gradual decline toward day 12, yet it remained the highest among all treatments. This indicates that salicylic acid acts as a potent elicitor of *PPO* activity, likely enhancing plant defense responses. Citric acid also stimulated *PPO* expression, reaching its maximum on days 4 and 8 before declining, while benzoic, malic, and acetic acids produced moderate increases that peaked around the same period. These results suggest that these acids trigger temporary but meaningful defense-related responses. In contrast, lactic acid showed weak induction, with expression levels consistently lower than those of the positive control throughout the experimental period, implying minimal influence on *PPO* activation. Overall, most acid treatments caused a progressive increase in *PPO* expression until day 4 or 8, followed by a decrease on day 12, reflecting a short-term activation of defense pathways. When compared to the POD (peroxidase) expression pattern with that of PPO, a similar transient response trend is observed; however, *PPO* induction by salicylic acid was stronger and more sustained than that of *POD*. This suggests that while both enzymes participate in plant defense, *PPO* may respond more robustly to salicylic acid treatment, highlighting its prominent role in the induced resistance mechanism.

### In Silico **analysis**

#### Protein models validation

The quality of the target protein models was assessed using Ramachandran plot and Verify3D analyses. The Verify3D results further confirmed the reliability of the predicted structures, with all models achieving an average 3D–1D score of ≥ 0.1, indicating high overall accuracy. The Ramachandran plots for all protein models are presented in Table S1, demonstrating that the majority of residues occupy favored and allowed regions, thereby confirming the structural reliability and suitability of these models for subsequent molecular docking studies.

#### In silico molecular docking of organic acids against target protein models

Among the tested organic acids, **salicylic** acid and benzoic acid exhibited the strongest binding affinities toward all examined protein targets, as revealed by molecular docking analysis. Both compounds showed high affinity for several *M. incognita* parasitic proteins, Table 6. In particular, salicylic acid displayed the lowest affinity energy with NAD(P)H oxidase (–4.54 kcal/mol), forming a hydrogen bond with the GLN816 residue, Tables 7 and 8. while **benzoic** acid interacted with the same protein (–4.39 kcal/mol) through hydrogen bonding with ARG770, Tables 6 and 7 and Fig. S3. Furthermore, both acids demonstrated notable binding energies with other target proteins, forming hydrogen bonds with various amino acid residues. These interactions suggest that salicylic and benzoic acids may play a significant role in modulating protein activity, thereby exhibiting potential nematicidal effects against *M. incognita*.

In addition, **malic acid** and **citric acid** also showed appreciable binding affinities with several protein targets. Notably, both exhibited strong docking energies with putative aspartyl protease (–4.34 and − 4.71 kcal/mol, respectively). Lactic acid displayed the weakest overall binding affinities among the tested organic acids, Tables 6 and 7 and Figs. S6, S4. However, Lactic acid showed the lowest affinity toward the target proteins, Tables 6 and 7 and Fig. S5.

For comparison, oxamyl (Vydate)—a commercially used nematicide—was included as a reference compound. Docking analysis revealed that oxamyl interacted with multiple protein targets but generally exhibited lower binding affinities than the organic acids, Tables 6 and 7 and Fig. S7. The docked complexes showed hydrogen bonding with backbone and side-chain residues within the binding sites, alongside other non-covalent interactions such as carbon–hydrogen, alkyl, π–alkyl, and π–sulfur contacts. These findings collectively underscore the pharmacological potential of organic acids in modulating nematode protein activity and suggest their possible use as natural alternatives to synthetic nematicide.

**Table 6:** interaction affinity score ( kcal/mol), RMSDValue (Å) of Molecular interactions between organic acids with the active-site residues of target proteins.

Target	Acetic acid	Benzoic acid	Citric acid	Lactic Acid	Malic acid	Salicylic acid	oxamyl
							
	interaction affinity score ( kcal/mol), RMSDValue (Å)						
Cytochrome c oxidase subunit 1	-3.33, 1.01	-4.14, 1.40	-4.62, 1.31	-3.83, 1.45	-4.19, 1.31	-4.25, 0.48	-5.73, 1.38
Putative aspartyl protease	-3.23, 0.89	-4.15, 0.89	-4.71, 1.82	-3.81, 1.28	-4.34, 1.26	-4.30, 1.64	-5.32, 1.50
Prefoldin-2	-3.02, 1.20	-3.92, 1.01	-3.87, 1.56	-3.49, 1.73	-3.80, 1.54	-4.29, 1.42	-4.90, 1.08
NAD(P)H oxidase	-3.62, 0.69	-4.39, 0.78	-4.56, 0.79	-3.02, 0.75	-4.08, 1.56	-4.54, 1.05	-5.81, 1.42
Venom allergen-like protein	-3.66, 1.18	-4.20, 1.67	-3.23, 1.58	-3.84, 1.13	-3.95, 2.85	-4.36, 1.71	-4.83, 0.97
Protein disulfide-isomerase	-3.01, 0.83	-3.71, 1.06	-3.90, 3.70	-3.54, 1.43	-3.70, 1.31	-4.08, 3.24	-5.05, 1.14

**Table 7:** Interactions between organic acids and the active-site residues of target proteins.

Target	Acetic acid	Benzoic acid	Citric acid	Lactic acid	Malic acid	Salicylic acid	Oxamyl
	(interactions), (Hydrogen bonds), (amino acids involved in Hydrogen bonds), distance of hydrogen bond Å)						
Cytochrome c oxidase subunit 1	(Van der Waals), No	(Carbon hydrogen, alkyl), No	(Carbon hydrogen), Yes, (GLY105), (1.98)	(carbon hydrogen and alkyl), No	(Carbon hydrogen), No	(carbon hydrogen and alkyl), No	(Carbon hydrogen, alkyl, pi alkyl), No
Putative aspartyl protease	(Carbon hydrogen and attractive charge), yes, (HIS50), (2.3)	(Carbon hydrogen, alkyl, attractive charge, and Pi alkyl), yes, (HIS50, TYR195), (2.8, 2.4)	(Carbon hydrogen, attractive charge), yes, (TYR195, ARG30, GLY28), (2.2, 2.5, 2.8)	(Carbon hydrogen, alkyl, attractive charge, and Pi alkyl), yes, (TYR195, ASP83, TYR46), (2.1, 2.2, 2.8)	(Carbon hydrogen, alkyl, attractive charge), yes, (TYR195, TYR46, ARG30) (1.85, 2.3, 2.5)	(alkyl, attractive charge, and Pi alkyl, metal acceptor, covalent bond), yes, (TYR195, TYR82, ARG30, HIS50), (2.1, 1.8, 2.1, 2.7)	(Carbon hydrogen, alkyl, pi alkyl, sulfur x), yes, (ARG30, TYR82, TYR46 ((2.7,2.1), 1.9, 3.0)
Prefoldin-2	(Van der Waals, Carbon hydrogen), yes, (ARG123), (2.1)	(Van der Waals, Pi alkyl), yes, (ARG123), (2.1)	(Carbon hydrogen), yes, (ARG123, HIS120, GLN21), (2.5, 2.0, 2.8)	(Van der Waals, Carbon hydrogen), No	(Carbon hydrogen), yes, (ARG123), (2.5)	(Pi alkyl), yes, ( ARG123, GLN21), (2.5, 2.6)	(Carbon hydrogen, alkyl, pi alkyl), yes, (LEU125, ILE122 (2.1, 2.3)
NAD(P)H oxidase	(Carbon hydrogen, attractive charge), yes, (ASN802), (2.2)	(Carbon hydrogen, attractive charge, and Pi alkyl), yes, (ARG770), (1.9)	(Carbon hydrogen, attractive charge), yes, (SER705, ARG722, ARG723), (2.0, 2.0, 2.8)	(Carbon hydrogen, attractive charge, salt bridge), yes, (ARG750, GLN753, PHE756), (2.0, 2.1, 2.9)	(Carbon hydrogen, attractive charge), yes, (PRO667), (2.1)	(Carbon hydrogen, attractive charge, salt bridge, Pi alkyl, pi cation), yes, (GLN816), (2.5)	(Carbon hydrogen, Pi alkyl, Alkyl, pi Sulfur), yes, (GLU700, ARG770, TRP697 (2.3, 2.4, 2.8)
Venom allergen-like protein	(Carbon hydrogen, salt bridge), yes, (CYC282, ARG117), (2.0, 2.1)	(---), yes, (ASN177, GLU88), (1.9, 2,3)	(Carbon hydrogen, pi anion), yes, (HIS79, GLU88, ASN177, GLY176, (2.8, 1.9, 2.7, 2.9)	(Carbon hydrogen, salt bridge, Pi alkyl), yes, (CYS282, ARG117, ASP283), ((1.9, 2,8), 2.1, 3.0)	(Carbon hydrogen, PI anion), yes, (HIS79,ASN177, GLU88) (2.7, 2.0, 1.9)	(---), yes, (GLU88, ASN177) (2.1, 1.9)	(Carbon hydrogen, Pi alkyl, Alkyl, pi Sulfur), yes, (TYR77, HIS142) (2.2, 2.5)
Protein disulfide-isomerase	(attractive charge), yes, (LYS306), (1.9)	(Carbon hydrogen, pi anion, salt bridge, Pi alkyl), yes, (LYS306), (2.2)	(salt bridge, attractive charge), yes, (GLU308, LYS307), (2.6, 2.1)	(Carbon hydrogen, alkyl, attractive charge), yes, (ASP292, ASP294, LYS307), (2.8, 1.9, 1.9)	(salt bridge, attractive charge, Carbon hydrogen), yes, (GLU301), (2.6)	(salt bridge, pi sigma, pi alkyl), No	(Carbon hydrogen, Alkyl, sulfur x), No.

**Table 8:** Molecular docking interaction of salicylic acid with the target protein active sides.

Target protein	Salicylic acid
Cytochrome c oxidase subunit 1	
Putative aspartyl protease	
Prefoldin-2	
NAD(P)H oxidase	
Venom allergen-like protein	
Protein disulfide-isomerase	

## Discussion

The present study provides a comprehensive evaluation of the nematicidal potential of selected organic acids and their associated effects on host defense responses, as evidenced by the expression profiles of defense-related genes quantified using real-time quantitative PCR (qPCR). Several low-molecular-weight organic acids have previously been reported to exhibit strong nematicidal activity against root-knot nematodes (RKNs). For example, butyric acid at concentrations of 0.1 and 1 mol L⁻¹ induced 100% mortality of *Meloidogyne hapla* and *M. incognita*^24^. Similarly, Bansal and Bajaj^25^ demonstrated that six volatile fatty acids significantly inhibited egg hatching, with propionic acid showing the highest efficacy, followed by acetic, caprylic, isobutyric, valeric, and butyric acids. These findings support the potential of organic acids as effective nematicidal agents and provide a comparative framework for interpreting the efficacy and molecular responses observed in the present study.

In this study, all tested organic acids (OAs) exhibited nematicidal activity against second-stage juveniles (J2s) of *Meloidogyne incognita*. Salicylic acid showed the highest efficacy, particularly during the first two days of exposure, even at the lowest concentration (0.01%), whereas benzoic and citric acids achieved 100% J2 mortality only after 72 h at higher concentrations. In contrast, lactic, malic, and acetic acids exhibited comparatively lower nematicidal activity, even at elevated concentrations. These findings indicate that the susceptibility of *Meloidogyne* juveniles to organic acids is compound-specific and influenced by physicochemical and biological factors.

Here, a critical question arises: what determines the susceptibility of juveniles to specific organic acids compared with others? Although all low molecular organic acid cause formation of multiple vacuoles in the body of dead nematodes, however, the differential susceptibility of *Meloidogyne* juveniles to organic acids is largely governed by acid dissociation constants (pKa), membrane permeability, molecular hydrophobicity, and their capacity to disrupt nematode metabolism and cuticle integrity. Organic acids that remain undissociated at physiological pH penetrate the nematode cuticle more efficiently, leading to intracellular acidification and metabolic disruption^26^. Aromatic acids such as salicylic and benzoic acids are more hydrophobic than aliphatic acids (acetic, lactic, malic, and citric acids), which enhances membrane partitioning and cellular uptake, thereby increasing cuticle permeability and intracellular toxicity^27^. Consequently, acids with optimal pKa values and higher lipid solubility exhibit greater nematicidal activity.

The observation that salicylic acid induced higher mortality at lower concentrations than at higher concentrations suggests a non-linear dose–response pattern consistent with hormesis. Hormesis is characterized by greater toxic effects at low doses, whereas higher doses induce adaptive or detoxification responses that reduce toxicity^28–30^. Salicylic acid is a weak organic acid whose ionization state and lipophilicity are concentration dependent; increased ionization at higher concentrations can reduce membrane permeability and intracellular accumulation, thereby lowering its effective toxic concentration^31^. Moreover, nematodes possess xenobiotic detoxification systems, including cytochrome P450 monooxygenases, glutathione S-transferases, and ATP-binding cassette transporters, which may be induced at high salicylic acid exposure and limit cellular damage^32^. Collectively, these factors likely explain the inverse dose–mortality relationship observed, reflecting hormetic toxicodynamic combined with physicochemical constraints on salicylic acid bioavailability and nematode stress-response mechanisms.

Chemotaxis also plays a critical role in determining nematode susceptibility to organic acids. Chemotaxis is a key behavioral mechanism that enables nematodes to orient and move in response to chemical cues, influencing feeding, metabolism, and signal perception^33,34^. Several organic acids, including acetic, lactic, succinic, and citric acids, have been reported as chemotactic cues affecting nematode orientation and development^35^. In the present study, mortality responses suggested a concentration-dependent behavioral pattern, where salicylic acid at low concentration and benzoic acid at high concentration were highly effective, while other acids exhibited a clear dose-dependent nematicidal effect. These observations support the concept that organic acids may act as attractants at low concentrations and as repellents or toxicants at higher concentrations. This pattern differs from Wuyts et al.^36^, who reported that salicylic acid strongly attracted *M. incognita* at low concentrations but showed nematicidal activity only at higher levels (LC₅₀ = 46 µg mL⁻¹), suggesting possible geographical or population-based variation in susceptibility. Furthermore, although lactic and acetic acids showed low toxicity in this study, Seo and Kim^29^ reported that their combination exhibited synergistic nematicidal activity, indicating that interactions among organic acids may significantly influence nematode mortality.

Plants produce a wide range of biologically active compounds with antagonistic effects against plant-parasitic nematodes, and several defense-related enzymes—including peroxidases (POD), polyphenol oxidase (PPO), phenylalanine ammonia lyase (PAL), and superoxide dismutase (SOD)—play critical roles in host responses during nematode infection^37^. Shravani^38^ reported the nematicidal properties of organic acids extracted from *Clonostachys rosea* against *Meloidogyne incognita* in vitro; however, their study lacked greenhouse or field validation and did not include transcriptomic analyses of treated and untreated nematode-infected plants.

This study focused on assessing the relative expression of polyphenol oxidase (PPO) and peroxidase (POD) genes because they play critical roles in plant innate immunity and induced defense responses. Polyphenol oxidase catalyzes the oxidation of phenolic compounds to quinones, which are highly reactive and toxic to invading pathogens and contribute to cell wall cross-linking and reinforcement^39^. While peroxidases are a large family of heme-containing oxidoreductase enzymes that utilize hydrogen peroxide (H₂O₂) to oxidize a wide range of substrates. POD genes encode enzymes that are central to plant defense, lignin polymerization, cell wall strengthening, and the regulation of oxidative stress^40^. Therefore, the expression levels of PPO and POD serve as important molecular markers for the activation of defense pathways in response to biotic stress.

From our findings, both genes exhibited maximum expression eight days after treatment, indicating that gene induction was closely associated with the timing of organic acid application and nematicidal activity. Similarly, Yang et al.^41^ reported differential expression patterns of lignin biosynthesis–related genes (PAL, C4H, HCT, and F5H) in nematode-infected tomato plants treated with oxalic acid, with temporal variation in transcript accumulation across different time points. Comparable temporal expression trends were observed in our study, suggesting early activation of defense mechanisms in response to nematode infection followed by a gradual decline as nematode-induced stress decreased due to the nematicidal effects of organic acids.

Results from the greenhouse experiment revealed that the application of organic acids—particularly salicylic acid (SA)—significantly reduced *Meloidogyne incognita* infection while simultaneously enhancing plant growth. The reduction in nematode propagation may be attributed, in part, to the strong acidity of organic acids, which can damage nematode cellular and tissue structures, disrupt osmoregulation, and cause intracellular fluid imbalance^42,29^. A significant increase in plant growth parameters is considered a primary indicator of induced systemic resistance (ISR) in nematode-infected plants treated with resistance-inducing agents (Sharaf et al. 2016) ^49^. In the present study, tomato plants treated with SA showed marked improvements in growth and productivity compared with other organic acid treatments. Although all applied organic acids improved plant growth, mainly due to their nematicidal effects, SA was more effective owing to two key factors: its higher efficacy in reducing nematode infection and its role as a key defense-related phytohormone that activates systemic acquired resistance (SAR)^43^. Similar outcomes have been reported previously; for example, soil inoculation with *Clonostachys rosea* strain IK726 reduced populations of plant-parasitic nematodes by 40–70% and significantly increased shoot length and biomass in carrot and wheat plants^44,45^. Previous studies have demonstrated that SA significantly reduces gall formation, egg masses, and nematode population densities through the activation of defense-related signaling pathways, indicating both direct and indirect nematicidal mechanisms^46–48^. In contrast, other OAs exerts nematicidal effects through acidity- and osmotic stress–mediated mechanisms, requiring longer exposure times to achieve comparable mortality with moderate inducing host defense responses. Therefore, the combined physicochemical properties and hormone-mediated defense signaling likely explain the superior efficacy of salicylic acid against root-knot nematodes.

Using a molecular docking approach, we investigated the nematicidal potential of organic acids (OAs) against several virulence-associated protein targets of *Meloidogyne incognita*, including cytochrome c oxidase subunit 1, prefoldin-2, putative aspartyl protease, protein disulfide isomerase 1, NAD(P)H oxidase, and venom allergen-like protein (Mi-VAP-2). All tested OAs exhibited moderate binding affinities toward these targets, which are known to play critical roles in nematode viability, development, and pathogenicity. Notably, salicylic acid, benzoic acid, malic acid, and citric acid showed higher binding affinities to the target sites compared with the commercial nematicide oxamyl, suggesting that these compounds may interfere with key physiological and metabolic processes in nematodes, such as cell penetration, intercellular migration, and redox signaling. The strong interactions observed between these OAs—particularly salicylic and benzoic acids—and proteins associated with nematode growth and virulence provide a plausible molecular mechanism underlying their nematicidal activity. These in silico findings are consistent with the experimental bioassay results, which demonstrated high nematicidal efficacy of these acids, supporting the hypothesis that OA–protein interactions contribute to reduced nematode viability and development. In a similar study, Shravani et al.^38^ employed molecular docking simulations to in silico evaluate the binding affinity of *Clonostachys rosea* bioactive compounds toward the same protein targets investigated in this study, and to predict the molecular mechanisms by which these compounds may modulate nematode viability through interference with protein function. However, a limitation of this study is the lack of biochemical and gene expression analyses to validate the predicted protein–ligand interactions. Future studies should incorporate enzymatic assays and transcriptomic analyses to confirm the molecular targets and pathways affected by these compounds. Although molecular docking is a widely used computational tool in pesticide development, it has inherent limitations, including dependence on the accuracy of protein structures, potential inaccuracies in scoring functions, assumptions of rigid ligand and protein conformations, limited conformational sampling, and the neglect of solvent effects. Despite these constraints, molecular docking remains a powerful preliminary approach for identifying and prioritizing candidate nematicidal compounds and provides a valuable framework for guiding subsequent experimental validation.

Collectively, the promising performance of organic acids as biocontrol agents in *in planta* experiments highlights their strong potential for integration into sustainable agricultural systems. Their ability to suppress nematode reproduction while simultaneously improving plant growth parameters indicates a dual role in pest management and crop productivity enhancement. In addition, the efficacy of organic acids—particularly salicylic acid at low concentrations—as environmentally friendly biocontrol agents. Their combined capacity to reduce nematode infestation and promote plant growth supports their potential as natural alternatives to synthetic nematicides within sustainable pest management strategies.

## Conclusion

This study highlights salicylic acid as the most effective and environmentally safe organic acid for managing *Meloidogyne incognita* infection in tomato plants. Among the six acids tested, salicylic acid at 0.01% achieved complete juvenile mortality and maximal inhibition of egg hatching, while significantly reducing root galling and nematode reproduction under greenhouse conditions. It also promoted plant growth and strongly upregulated defense-related genes (*POD* and *PPO*), indicating activation of systemic resistance. In silico molecular docking supported these findings, showing that salicylic and benzoic acids had the highest binding affinities to nematode pathogenicity-related proteins, suggesting interference with essential biological processes. Collectively, these results establish salicylic acid as a potent, low-cost, and eco-friendly alternative to synthetic nematicides, offering both direct nematode suppression and enhanced plant defense responses.

## Supplementary Information

Below is the link to the electronic supplementary material.

Supplementary Material 1

## Data Availability

All data generated or analysed during this study are included in this article.
