# Supplementary material for: Organic acids induce plant defense responses and suppress root-knot nematodes in tomato via molecular mechanisms and molecular docking insights
Source: Sci Rep. 2026 Jun 10;16:18012. doi: 10.1038/s41598-026-56502-9 (PMC13254392; doi:10.1038/s41598-026-56502-9)
Supplement: Supplementary file 1 — Supplementary Material 1 [file 41598_2026_56502_MOESM1_ESM.docx]

**Table (S1 ):** PROCHECK plot statistical analysis of protein target models

| **protein** | **PROCHECK plot statistics** | | | |
| --- | --- | --- | --- | --- |
|  | Most favored  regions | Additional Allowed regions | Generously allowed.  regions | Disallowed regions |
| Cytochrome c oxidase subunit 1 | 95.5% | 4.5% | 0% | 0% |
| Putative aspartyl protease | 89% | 11% | 0% | 0% |
| Prefoldin-2 | 97.2% | 2.2% | 0% | 0% |
| NAD(P)H oxidase | 91.6% | 7.8% | 0.4% | 0.1% |
| Venom allergen-like protein | 88.2% | 10.2% | 0.7 | 0.9% |
| protein disulfide-isomerase | 93.2% | 5.6% | 0.5% | 0.7% |

**Table (S2 ):** Molecular docking interaction of Acetic acid with the target protein active sides

|  | **Acetic acid** |
| --- | --- |
| **Cytochrome c oxidase subunit 1** | 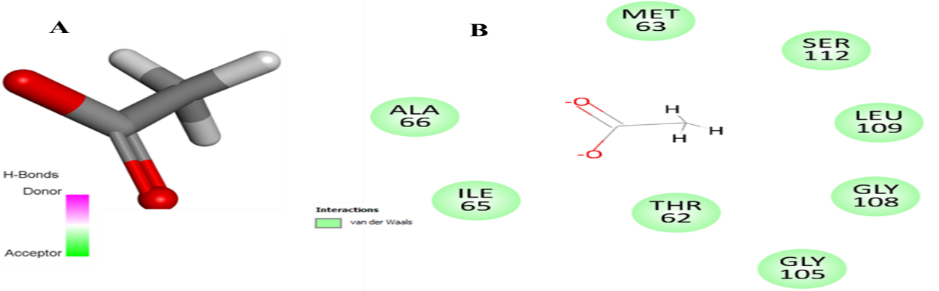 |
| **Putative aspartyl protease** | 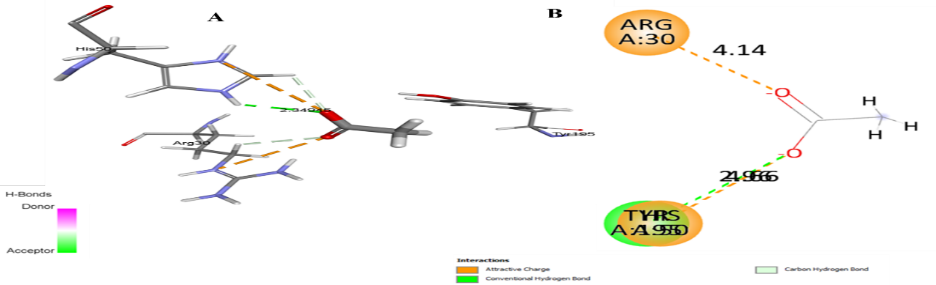 |
| **Prefoldin-2** | 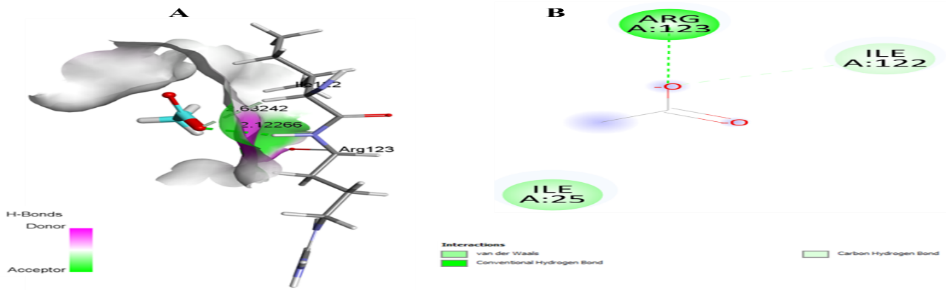 |
| **NAD(P)H oxidase** | 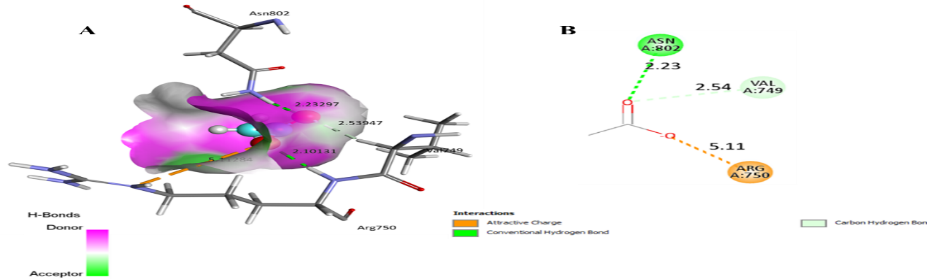 |
| **Venom allergen-like protein** | 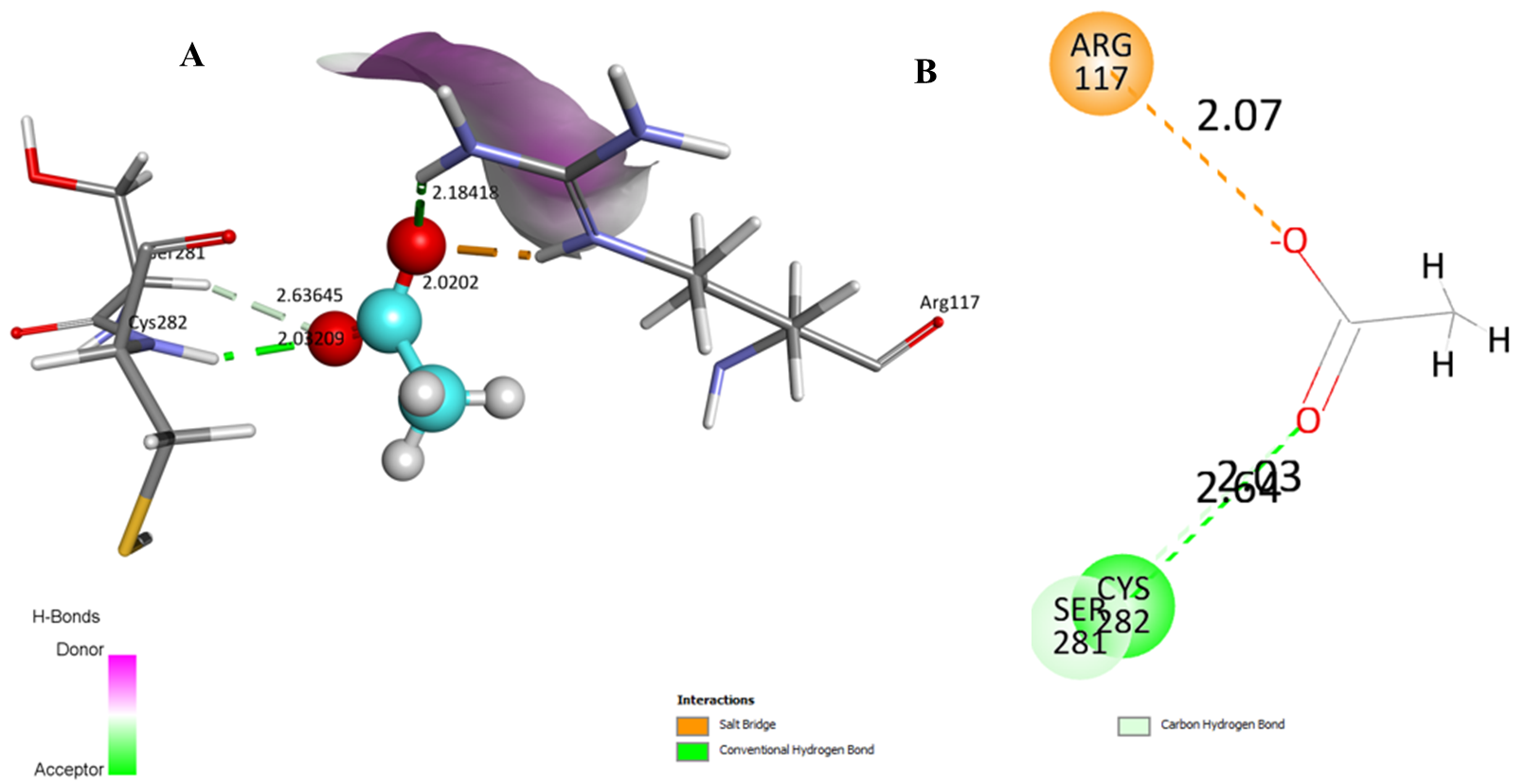 |
| protein disulfide-isomerase | 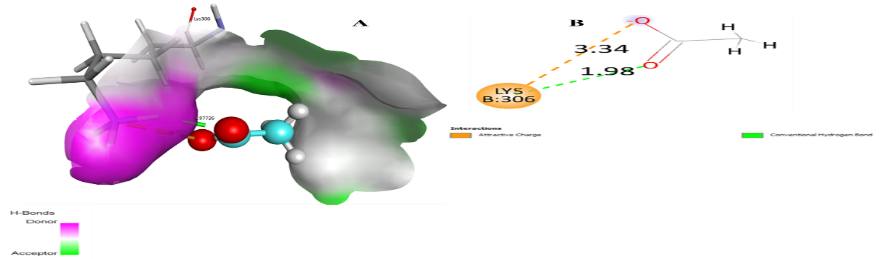 |

**Table (S3 ):** Molecular docking interaction of benzoic acid with the target protein active sites

|  | **Benzoic acid** |
| --- | --- |
| **Cytochrome c oxidase subunit 1** | 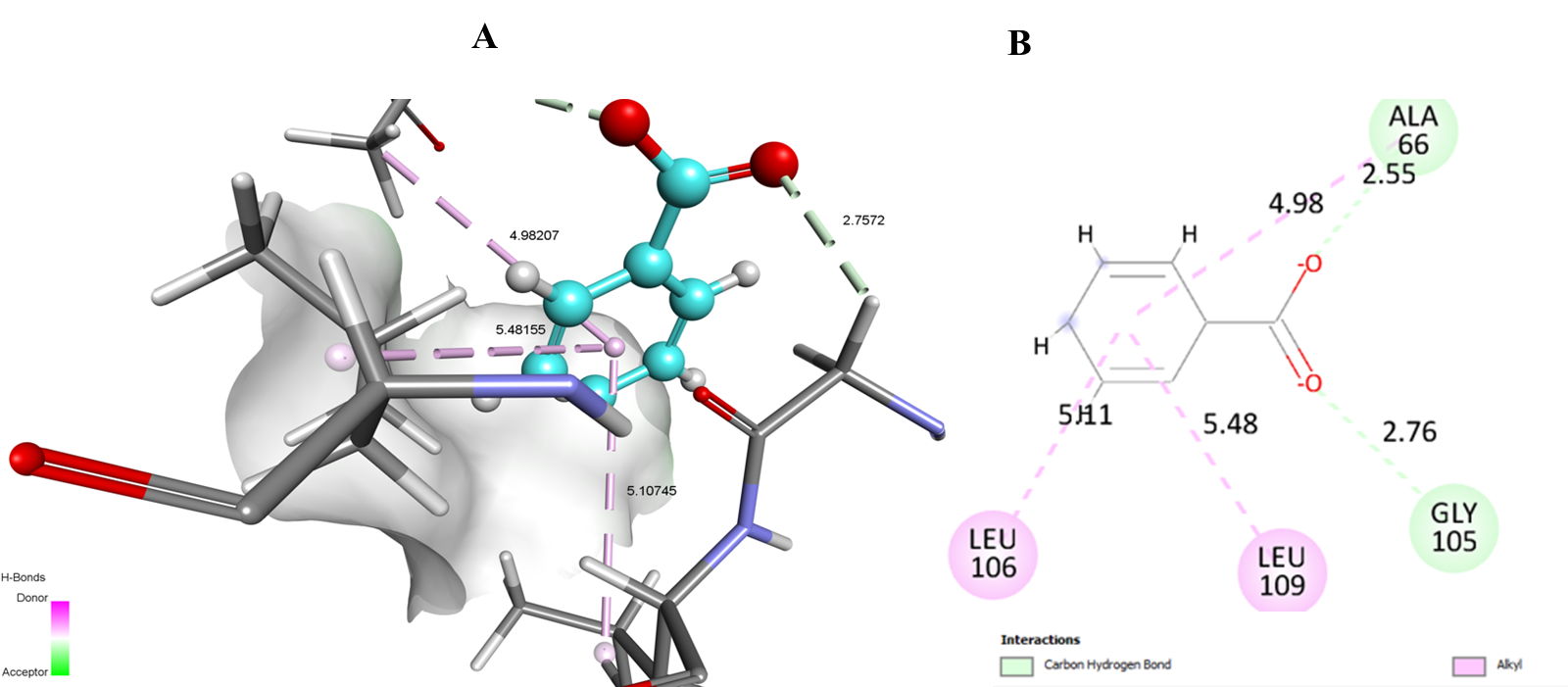 |
| **Putative aspartyl protease** | 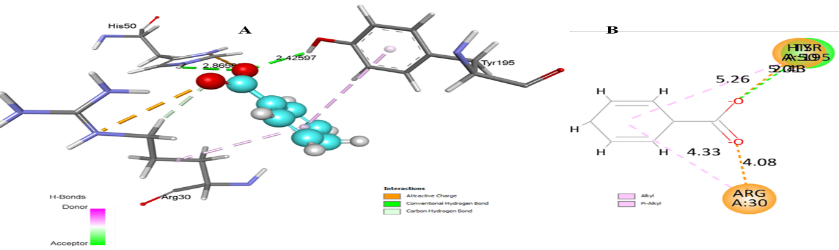 |
| **Prefoldin-2** | 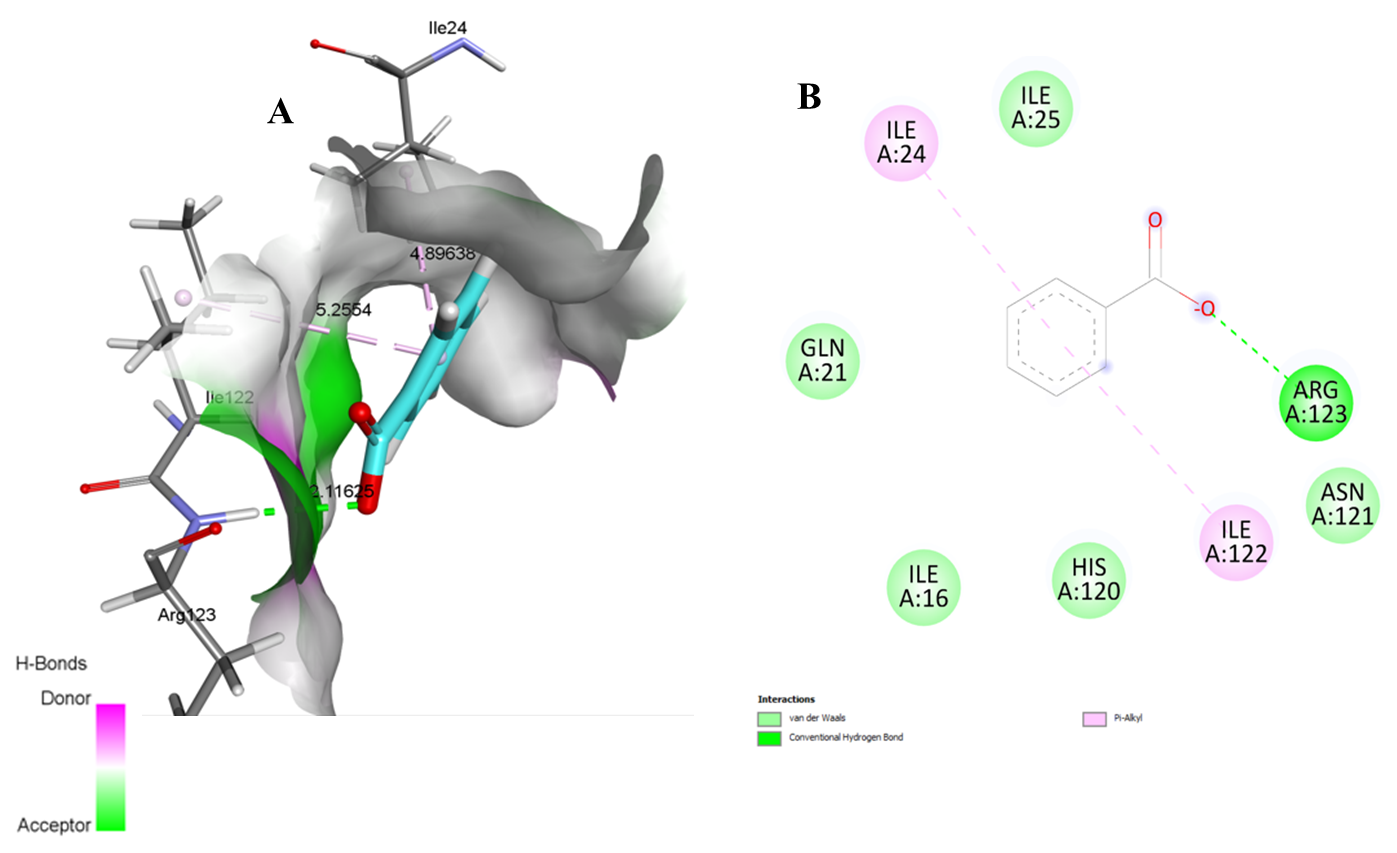 |
| **NAD(P)H oxidase** | 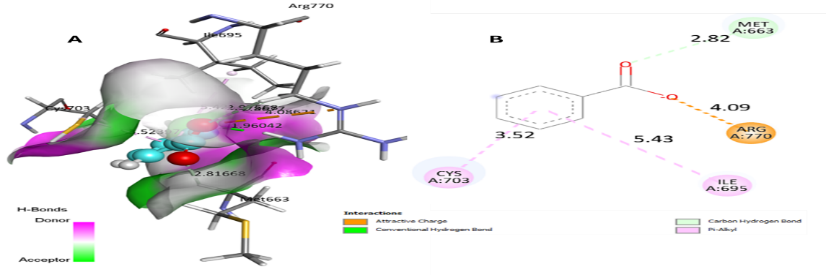 |
| **Venom allergen-like protein** | 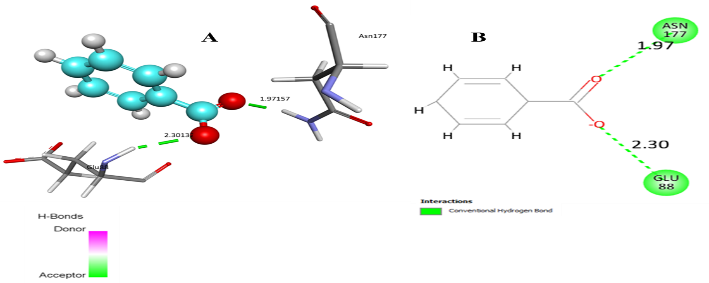 |
| **protein disulfide-isomerase** | 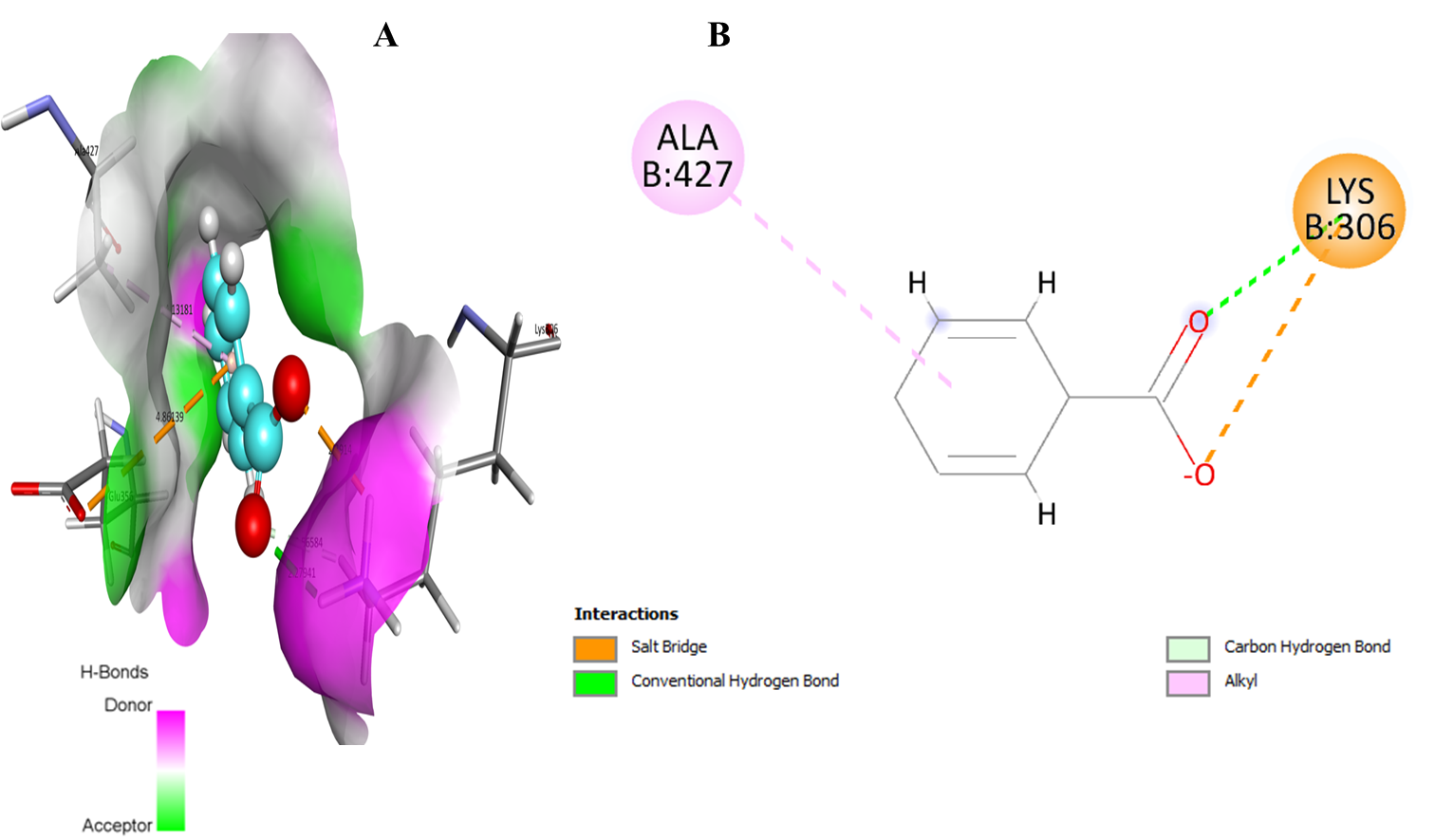 |

**Table (S4 ):** Molecular docking interaction of Citric acid with the target protein active sides

|  | **Citric acid** |
| --- | --- |
| **Cytochrome c oxidase subunit 1** | 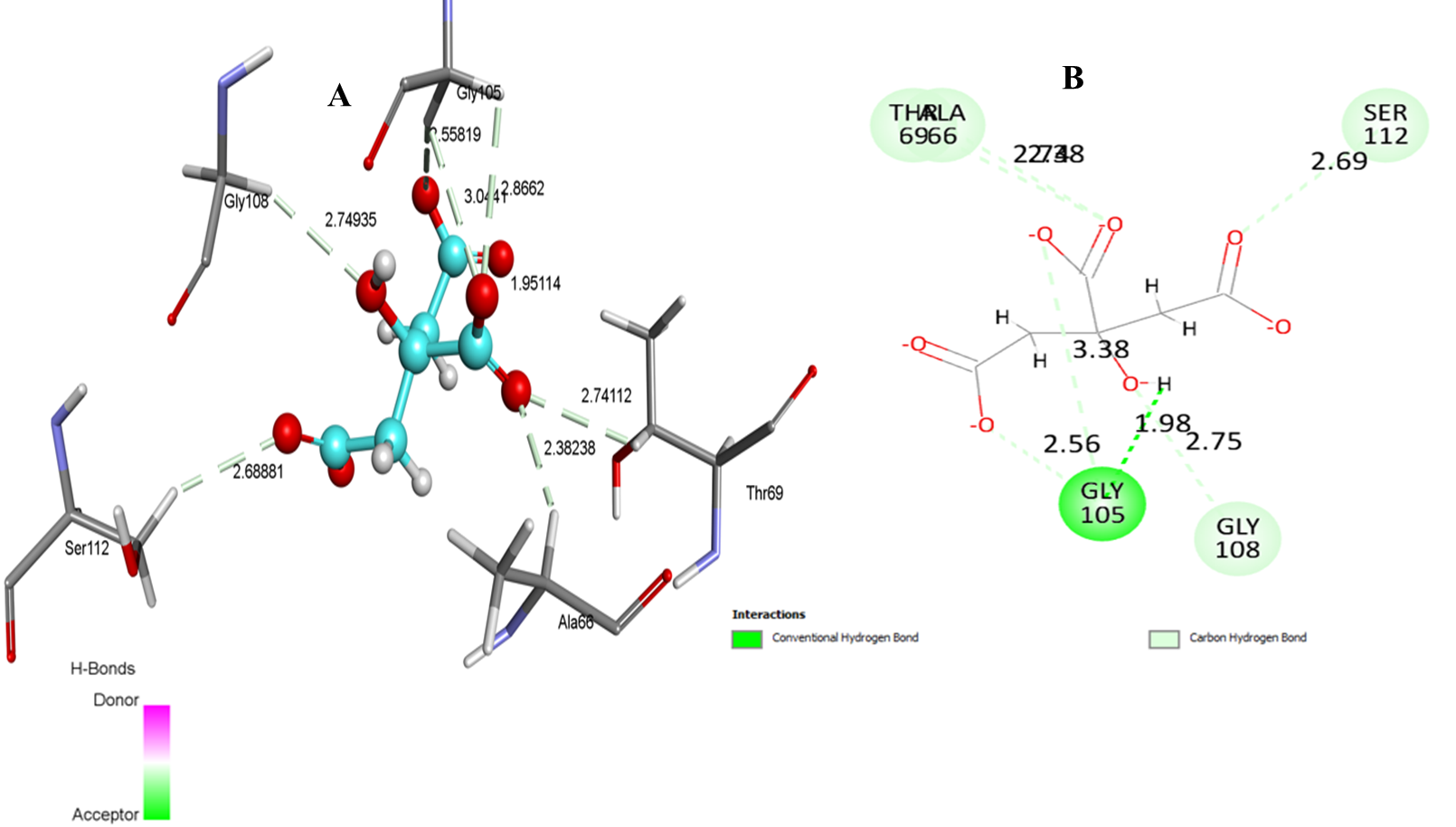 |
| Putative aspartyl protease | 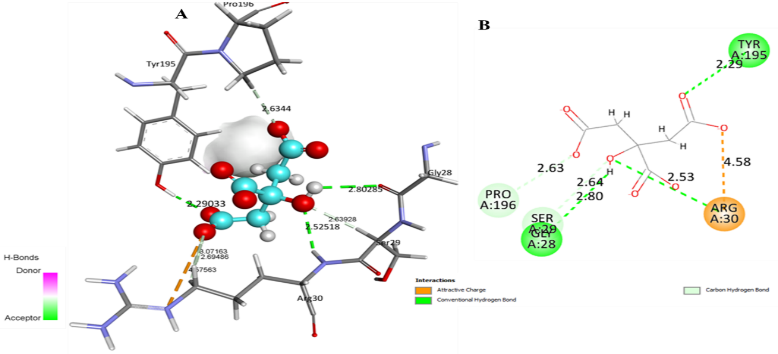 |
| **Prefoldin-2** | 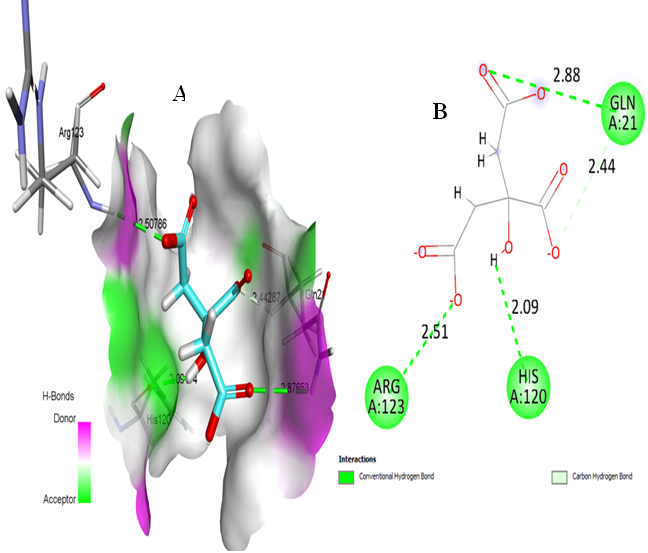 |
| **NAD(P)H oxidase** | 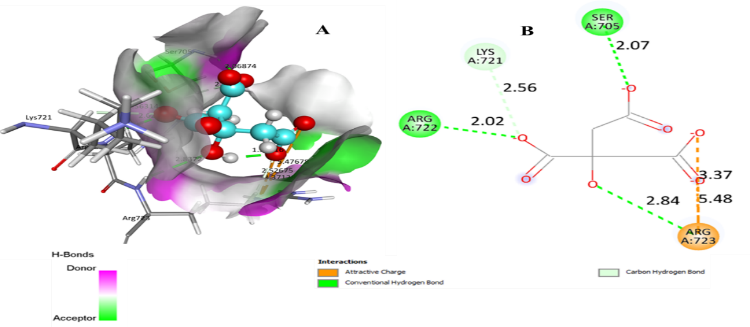 |
| **Venom allergen-like protein** | 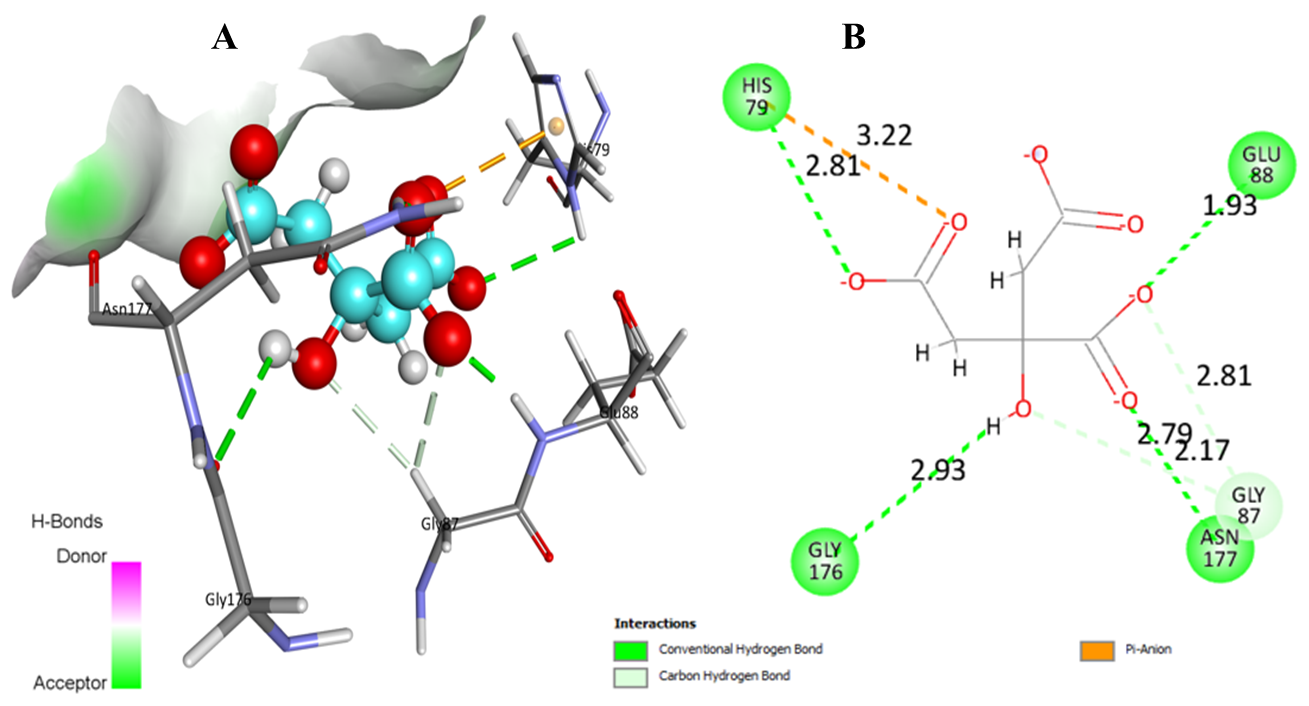 |
| **protein disulfide-isomerase** | 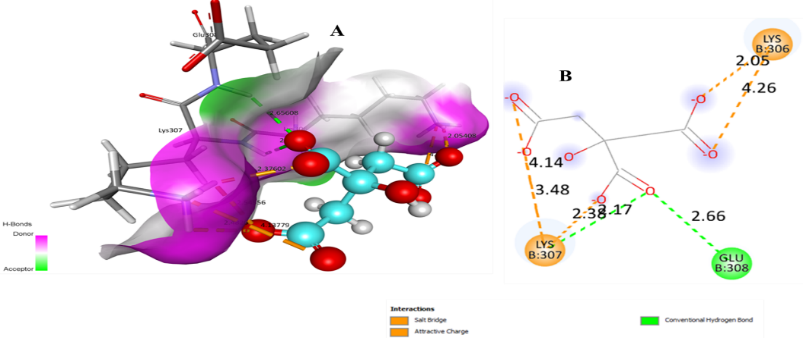 |

**Table (S5 ):** Molecular docking interaction of lactic acid with the target protein active sits

|  | **Lactic acid** |
| --- | --- |
| **Cytochrome c oxidase subunit 1** | 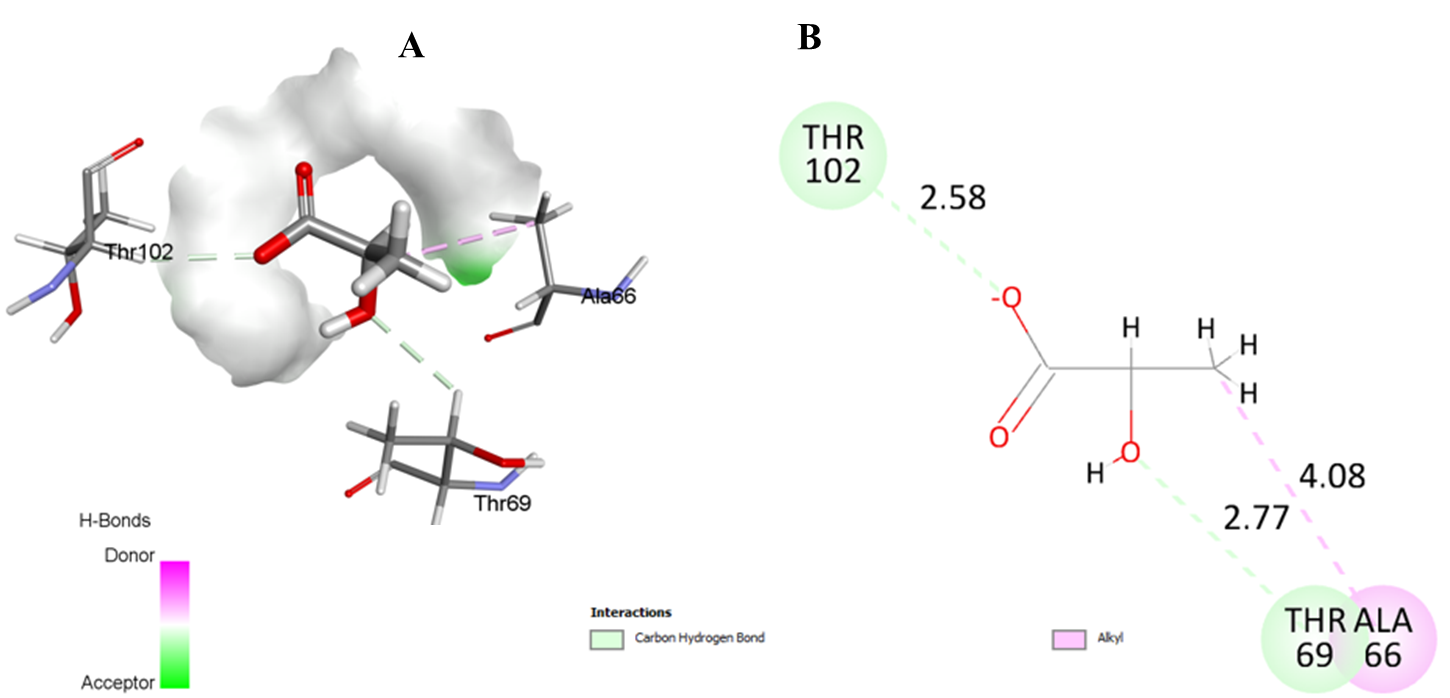 |
| Putative aspartyl protease | 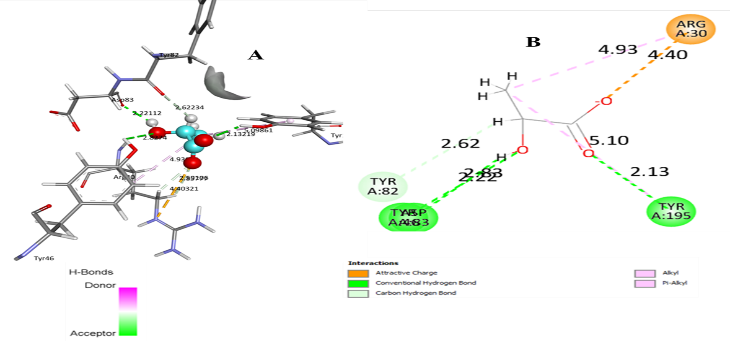 |
| **Prefoldin-2** | 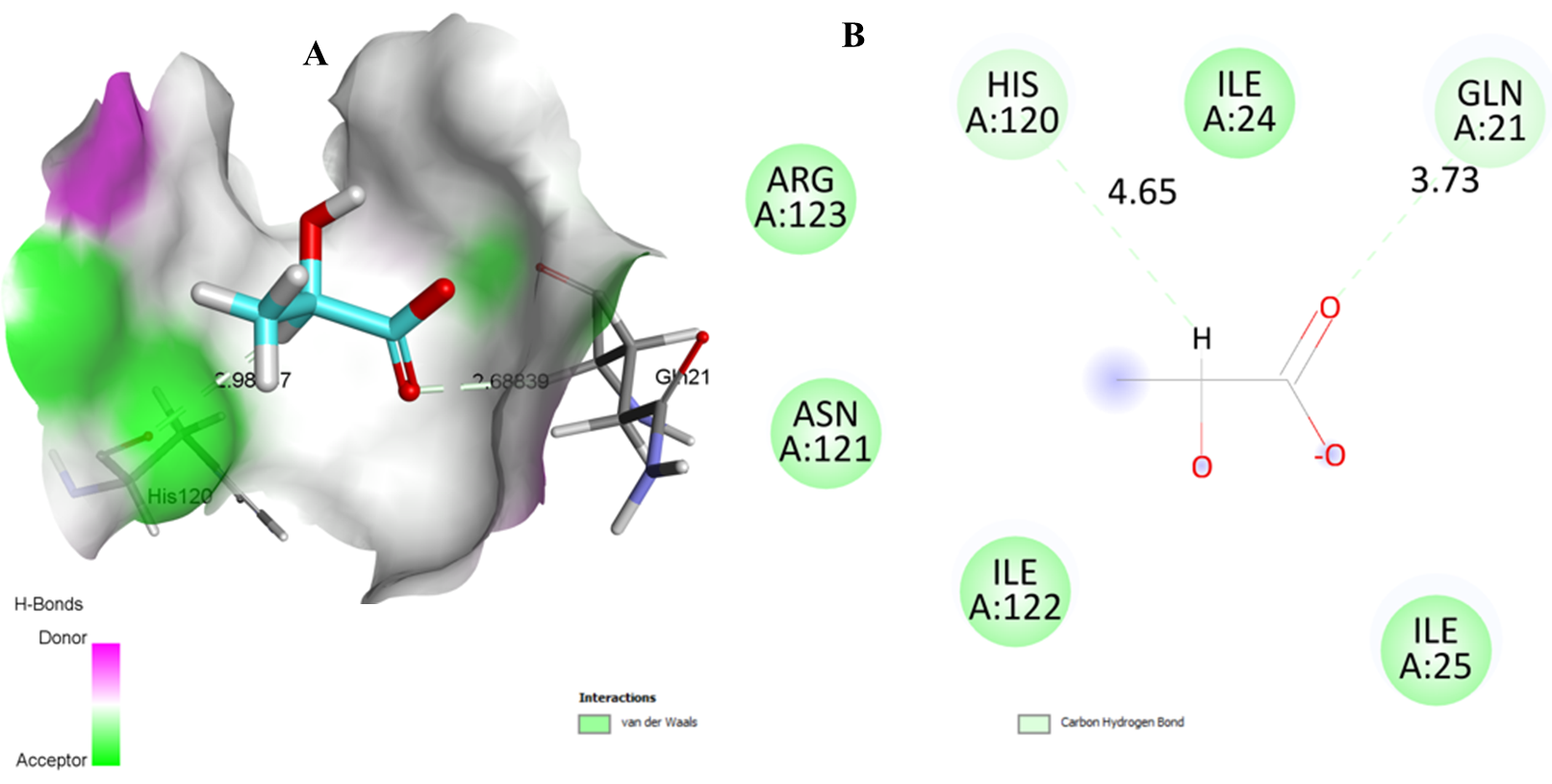 |
| **NAD(P)H oxidase** | 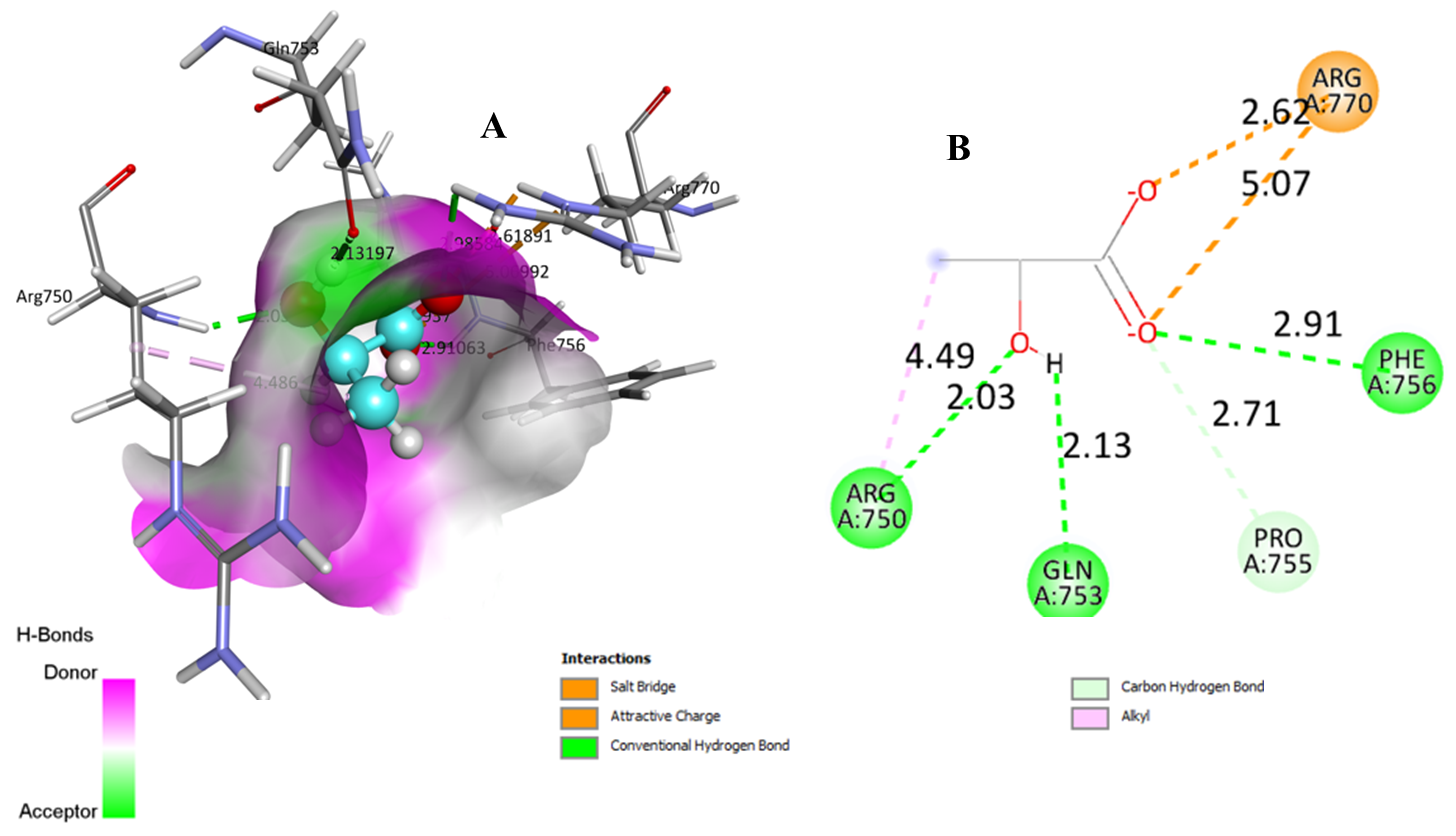 |
| **Venom allergen-like protein** | 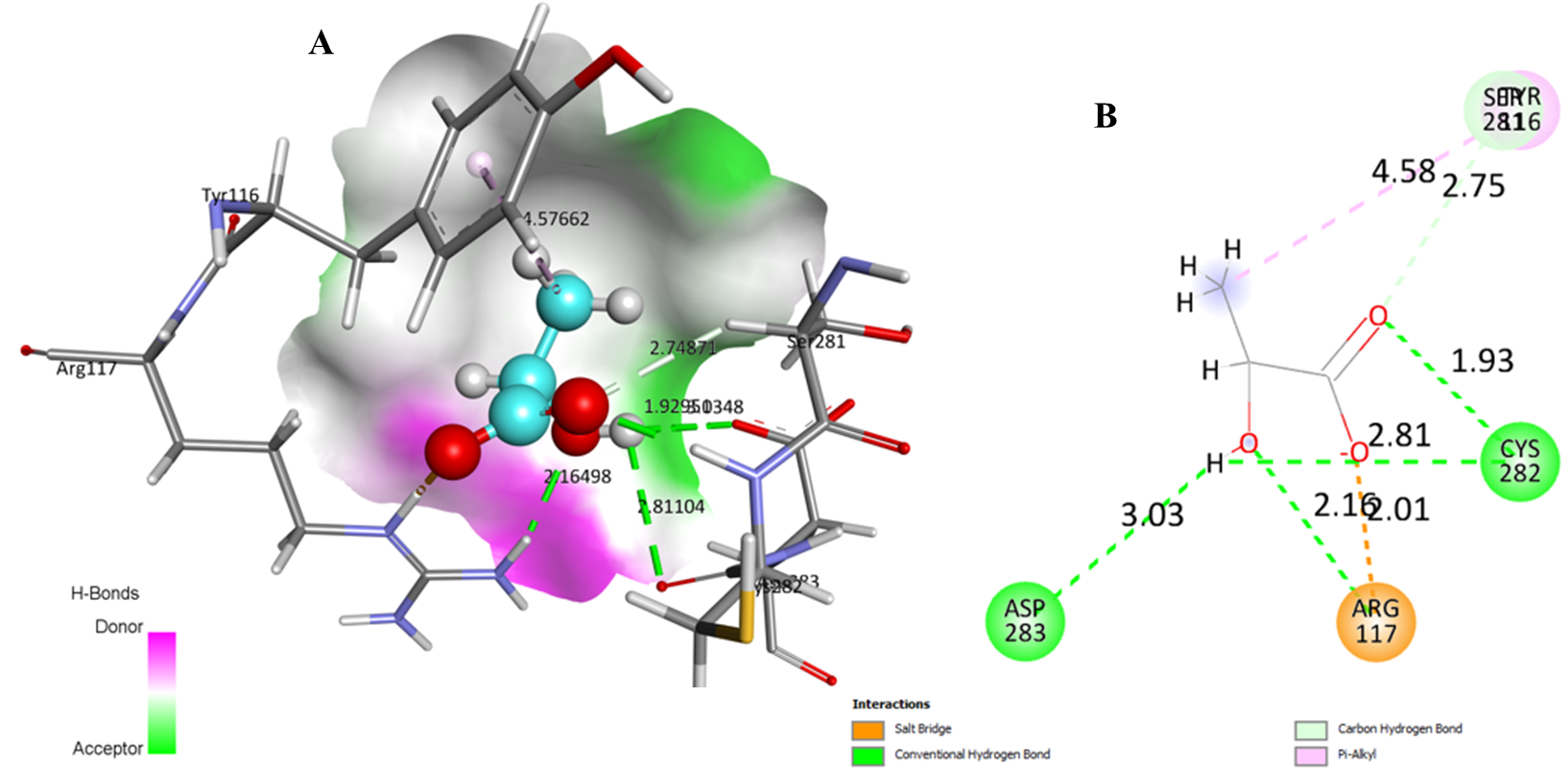 |
| **protein disulfide-isomerase** | 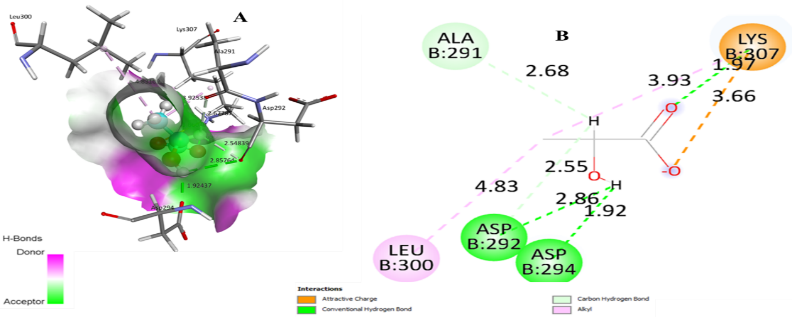 |

**Table (S6** **):** Molecular docking interaction of malic acid with the target protein active sites

|  | **Malic acid** |
| --- | --- |
| **Cytochrome c oxidase subunit 1** | 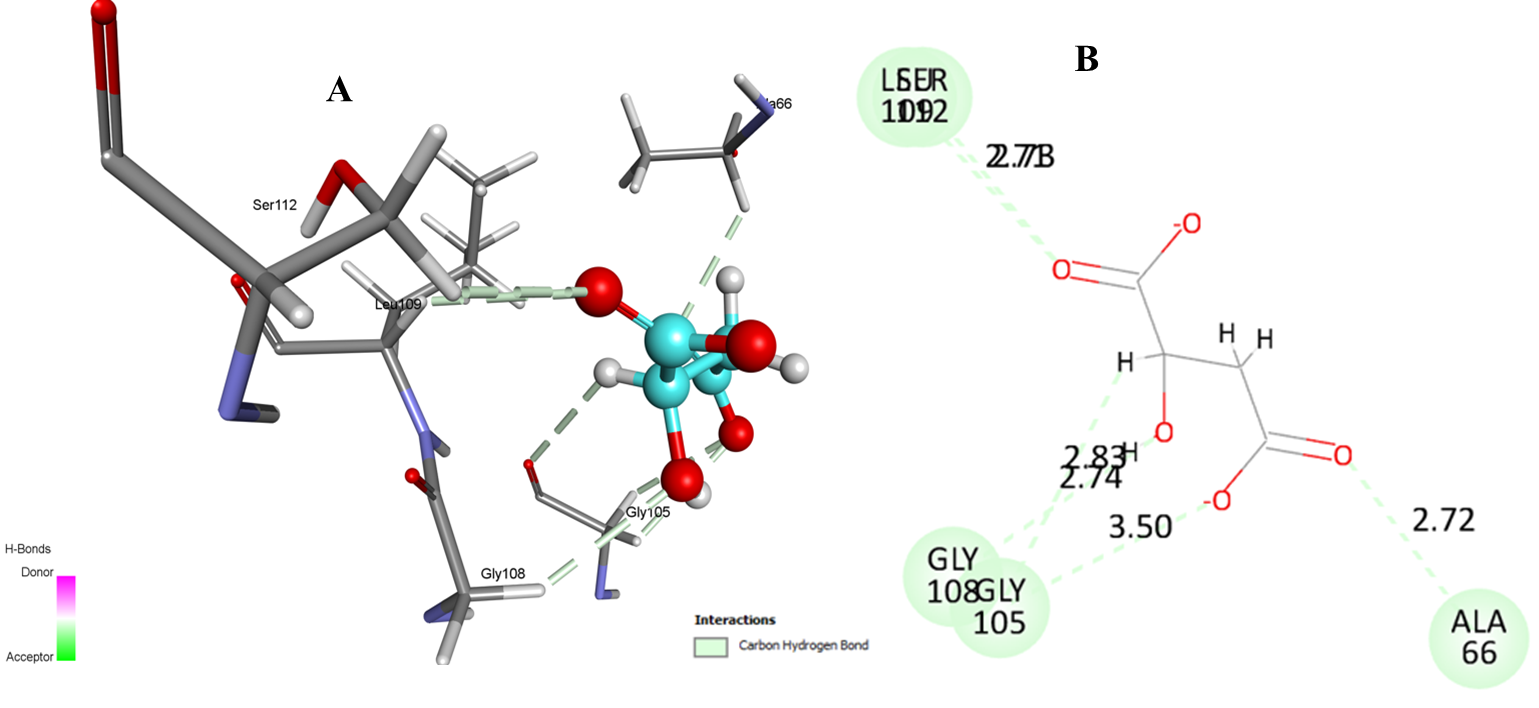 |
| **Putative aspartyl protease** | 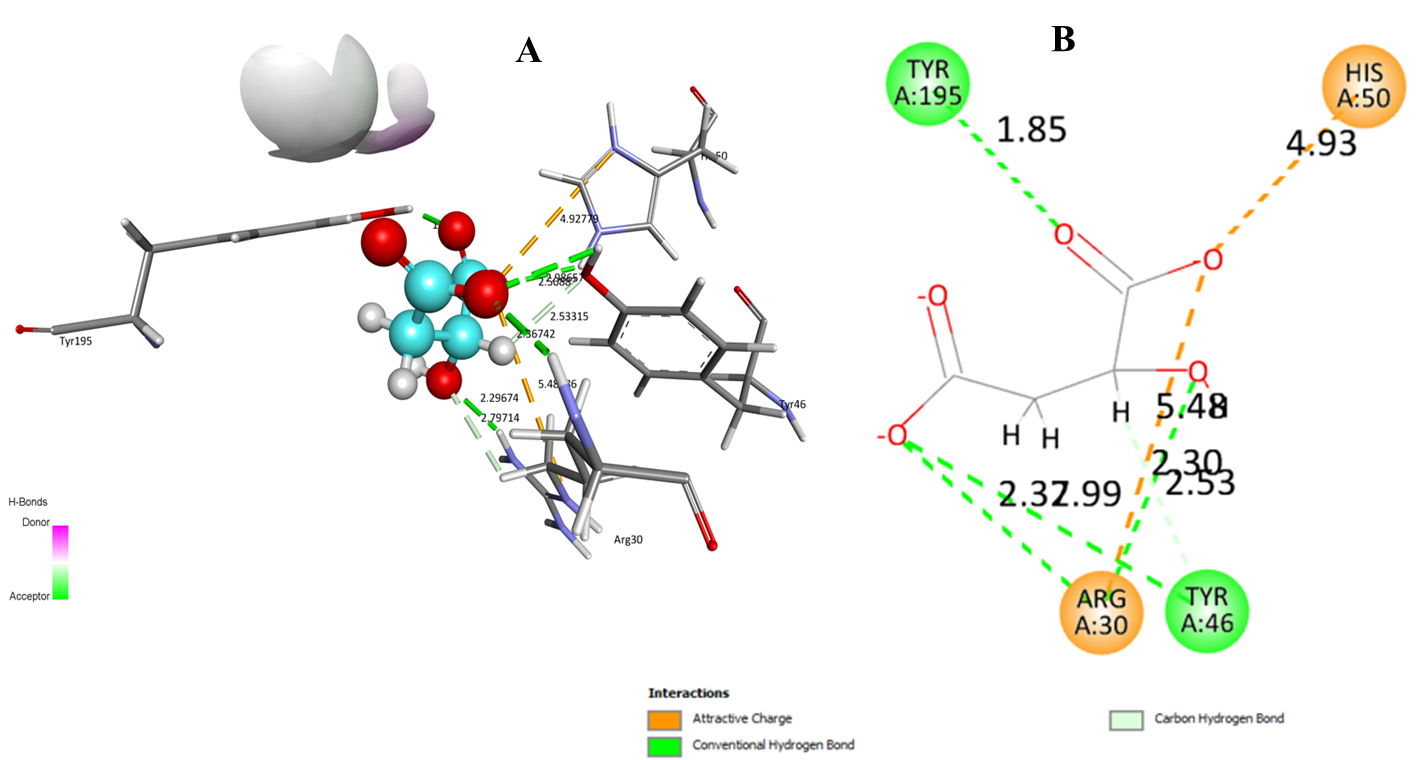 |
| **Prefoldin-2** | 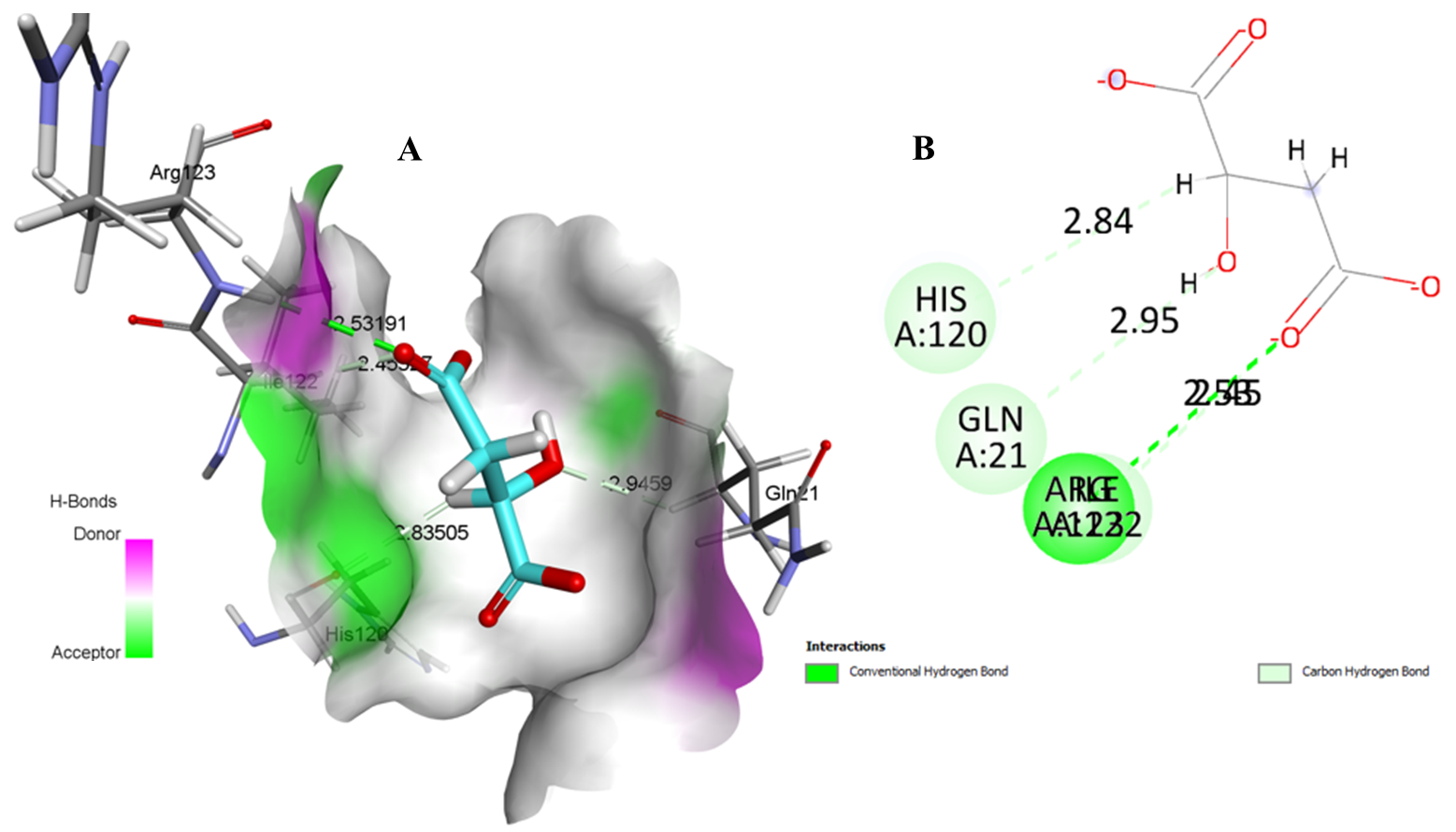 |
| NAD(P)H oxidase | 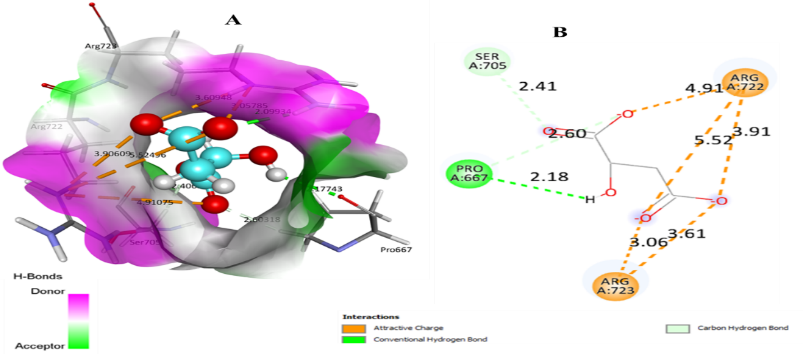 |
| **Venom allergen-like protein** | 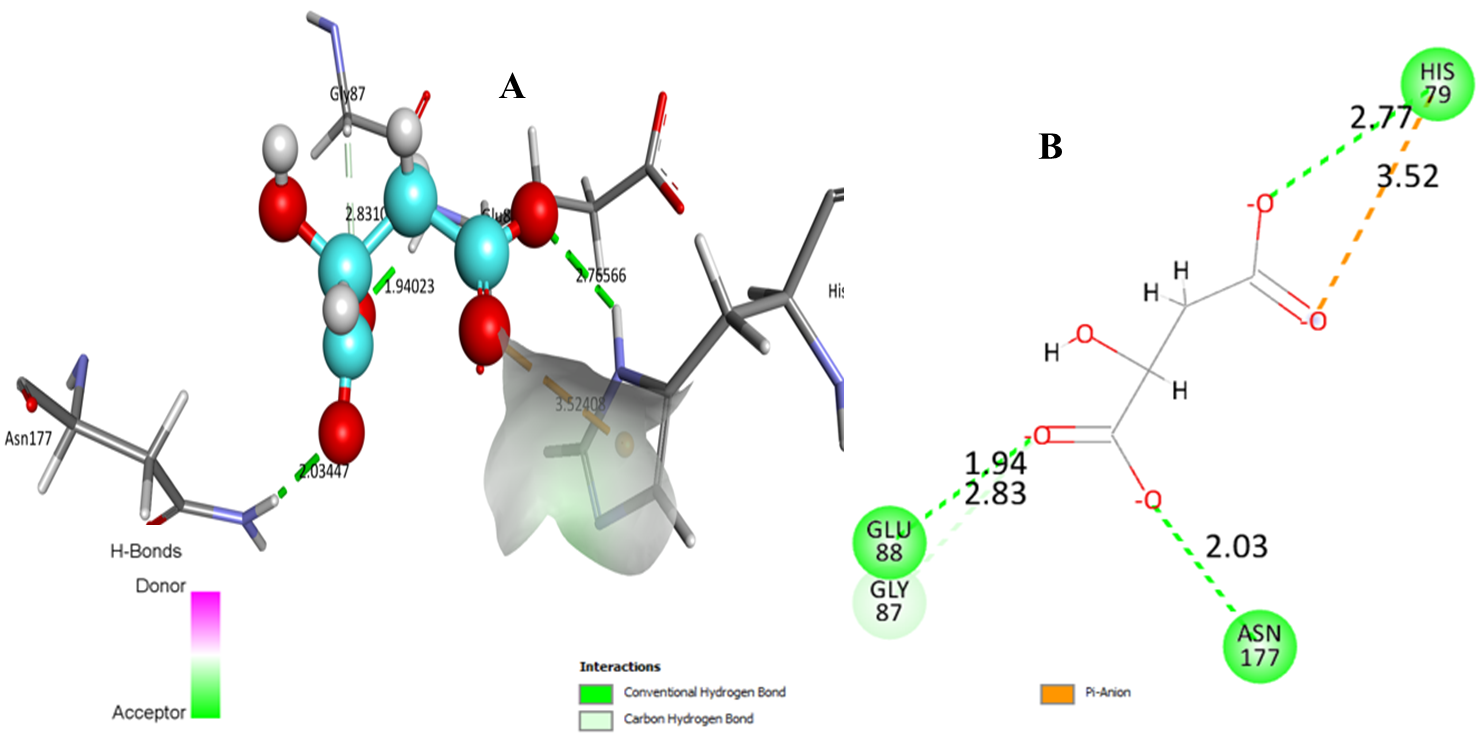 |
| **protein disulfide-isomerase** | 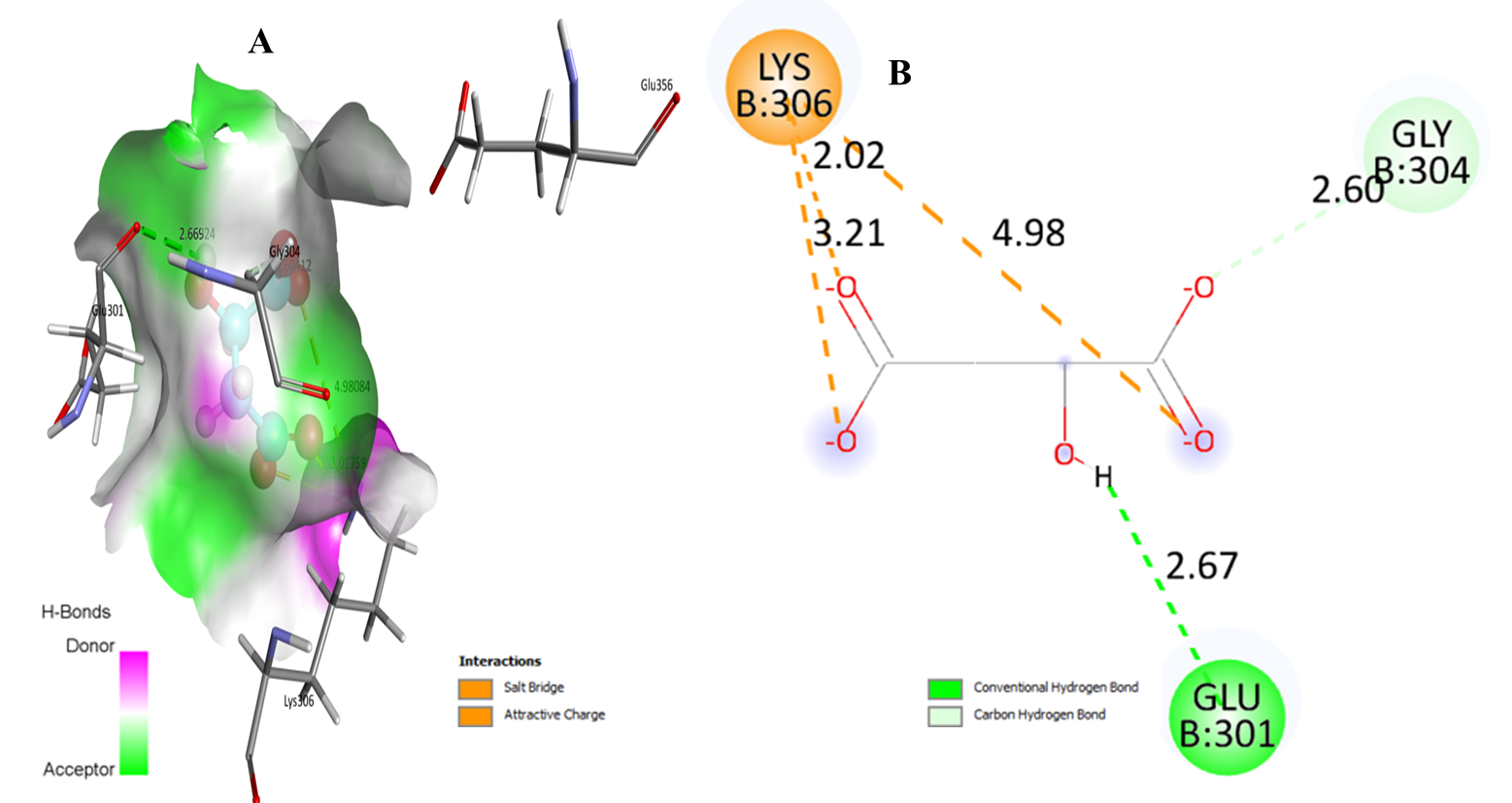 |

**Table (S7 ): ):** Molecular docking interaction of oxamyl with the target protein active sites

|  | **Oxamyl** |
| --- | --- |
| **Cytochrome c oxidase subunit 1** | 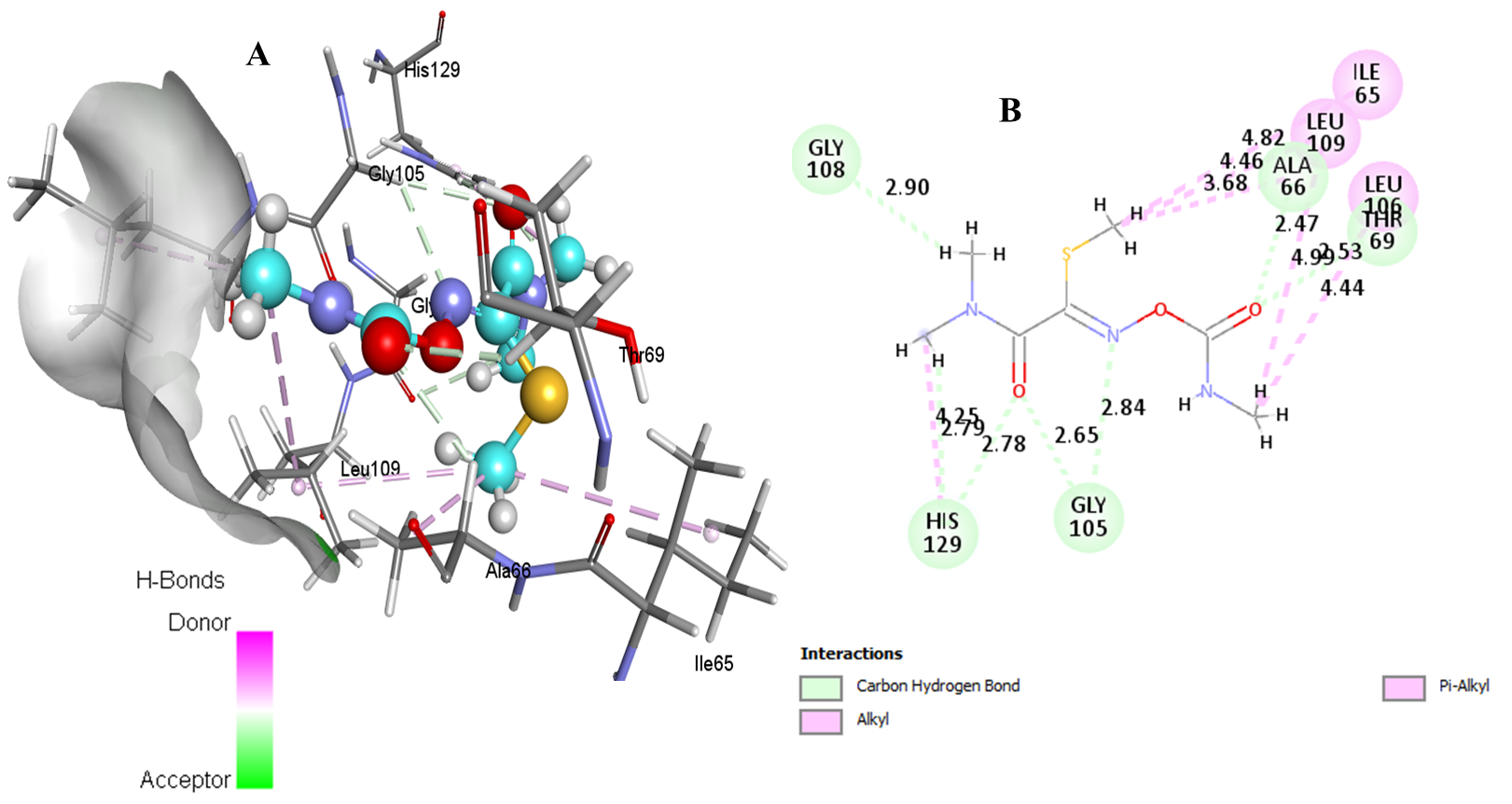 |
| Putative aspartyl protease | 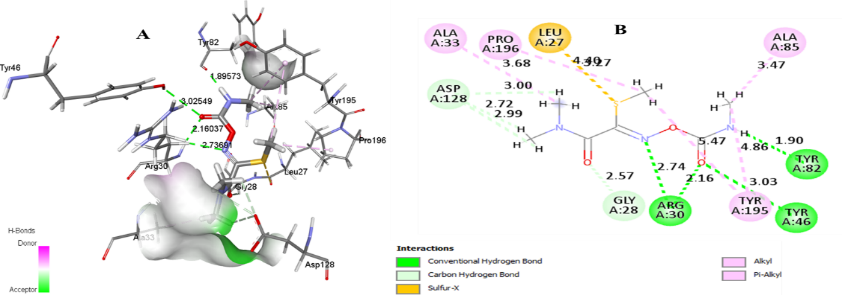 |
| **Prefoldin-2** | 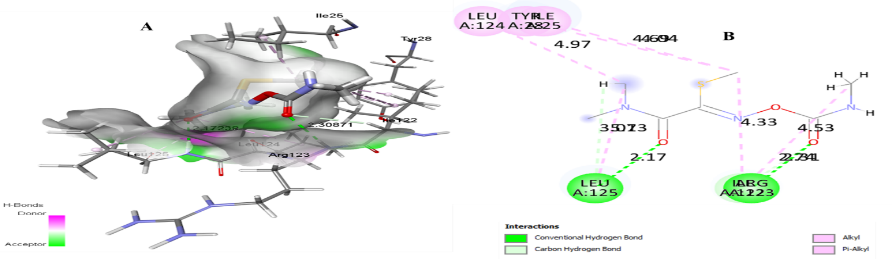 |
| **NAD(P)H oxidase** | 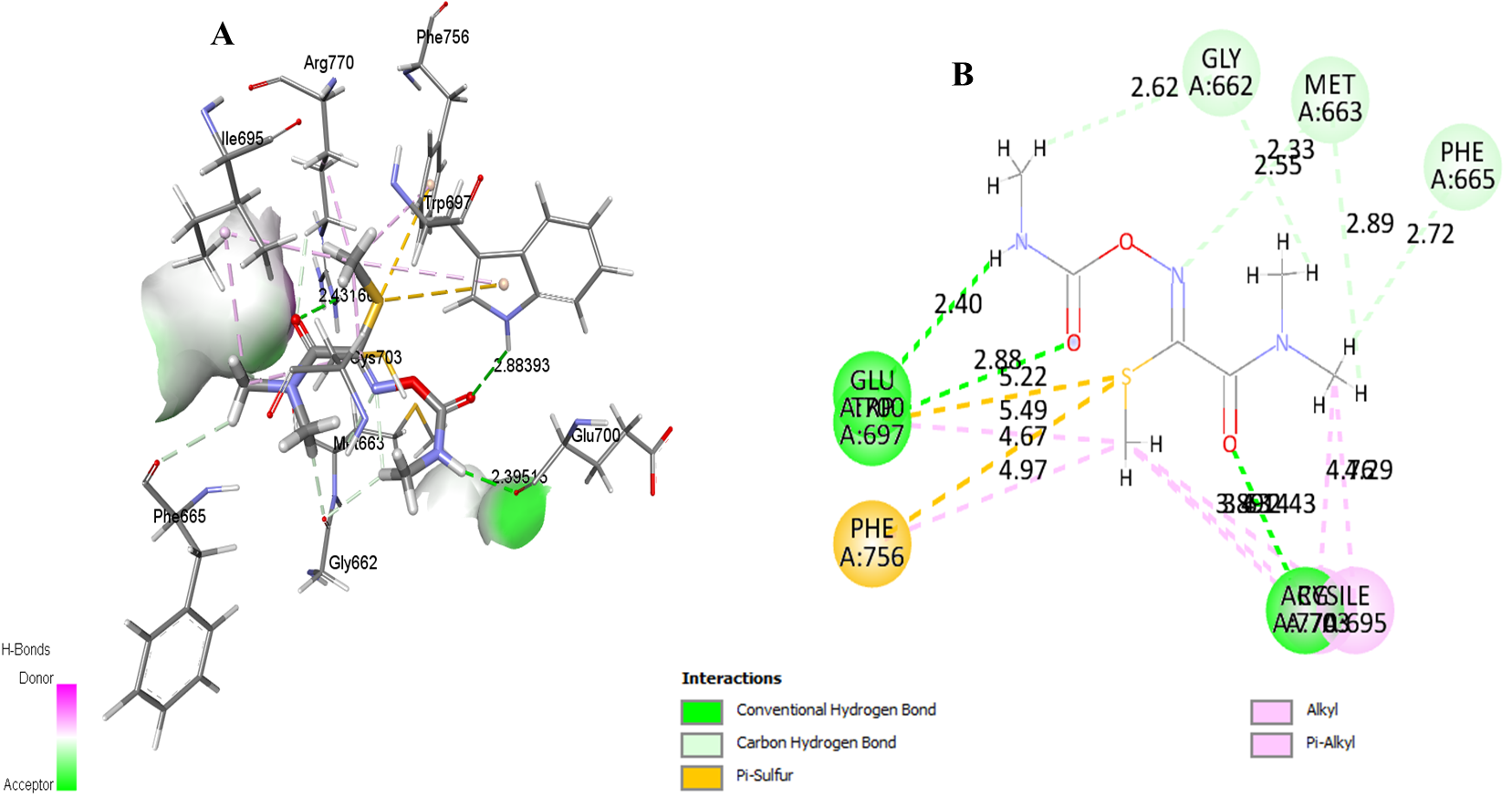 |
| **Venom allergen-like protein** | 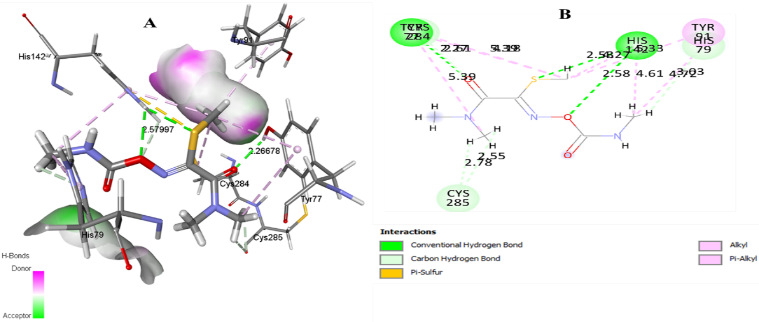 |
| **protein disulfide-isomerase** | 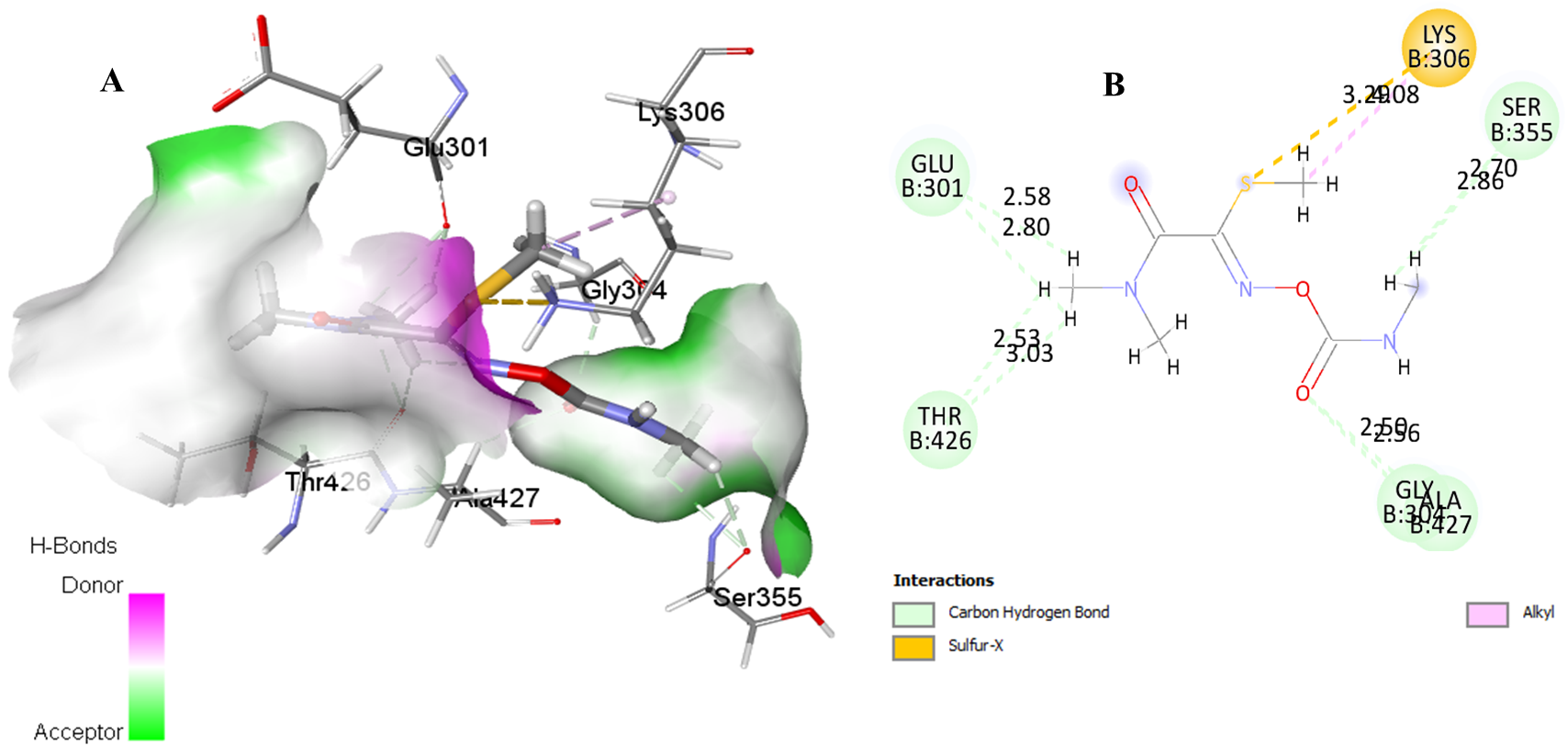 |
